# Experimental Evidence of Intrinsic Disorder and Amyloid Formation by the *Henipavirus* W Proteins

**DOI:** 10.3390/ijms23020923

**Published:** 2022-01-15

**Authors:** Giulia Pesce, Frank Gondelaud, Denis Ptchelkine, Juliet F. Nilsson, Christophe Bignon, Jérémy Cartalas, Patrick Fourquet, Sonia Longhi

**Affiliations:** 1 Laboratoire Architecture et Fonction des Macromolécules Biologiques (AFMB), UMR 7257, Aix Marseille University and Centre National de la Recherche Scientifique (CNRS), 163 Avenue de Luminy, Case 932, 13288 Marseille, France; giulia.pesce@univ-amu.fr (G.P.); frank.gondelaud@univ-amu.fr (F.G.); denis.PTCHELKINE@univ-amu.fr (D.P.); juliet.nilsson@univ-amu.fr (J.F.N.); christophe.bignon@univ-amu.fr (C.B.); jeremycartalas@orange.fr (J.C.); 2 INSERM, Centre de Recherche en Cancérologie de Marseille (CRCM), Centre National de la Recherche Scientifique (CNRS), Marseille Protéomique, Institut Paoli-Calmettes, Aix Marseille University, 27 Bvd Leï Roure, CS 30059, 13273 Marseille, France; patrick.fourquet@inserm.fr

**Keywords:** intrinsically disordered proteins/regions, small-angle X-ray scattering, innate immune response evasion, phase separation, fibrillation, biocondensates, Congo red and Thioflavin T binding assays, negative-staining electron microscopy, amyloid-like fibrils, viral proteins

## Abstract

Henipaviruses are severe human pathogens within the *Paramyxoviridae* family. Beyond the P protein, the *Henipavirus* P gene also encodes the V and W proteins which share with P their N-terminal, intrinsically disordered domain (NTD) and possess a unique C-terminal domain. *Henipavirus* W proteins antagonize interferon (IFN) signaling through NTD-mediated binding to STAT1 and STAT4, and prevent type I IFN expression and production of chemokines. Structural and molecular information on *Henipavirus* W proteins is lacking. By combining various bioinformatic approaches, we herein show that the *Henipaviruses* W proteins are predicted to be prevalently disordered and yet to contain short order-prone segments. Using limited proteolysis, differential scanning fluorimetry, analytical size exclusion chromatography, far-UV circular dichroism and small-angle X-ray scattering, we experimentally confirmed their overall disordered nature. In addition, using Congo red and Thioflavin T binding assays and negative-staining transmission electron microscopy, we show that the W proteins phase separate to form amyloid-like fibrils. The present study provides an additional example, among the few reported so far, of a viral protein forming amyloid-like fibrils, therefore significantly contributing to enlarge our currently limited knowledge of viral amyloids. In light of the critical role of the *Henipavirus* W proteins in evading the host innate immune response and of the functional role of phase separation in biology, these studies provide a conceptual asset to further investigate the functional impact of the phase separation abilities of the W proteins.

## 1. Introduction

The Nipah and Hendra viruses (NiV and HeV) are zoonotic pathogens gathered in the *Henipavirus* genus within the *Paramyxoviridae* family in the *Mononegavirales* order. In human beings they cause severe acute respiratory syndrome, generalized vasculitis and lethal encephalitis. Their natural reservoir are fruit-eating bats of the *Pteropus* genus, the so-called flying foxes [1,2]. HeV came to light in 1994 in the Hendra suburb of Brisbane (Australia) as a new agent responsible for a sudden outbreak of an acute respiratory and neurologic disease within horses [1]. Exposure to body fluids and tissues or excretions of infected horses resulted in transmission to humans, with a high case fatality (~60%). NiV emerged in 1998 in Malaysia as the causative agent of an outbreak of disease in pigs and humans with 265 human cases of encephalitis and 105 deaths [1]. Since its first emergence, NiV reappeared in 2001 in Bangladesh (where outbreaks of encephalitis, with an average case fatality rate of 80%, have occurred almost annually) and successively in India and in Philippines. Owing to the wide distribution of bats and to the broad spectrum of susceptible animals, additional spillover events into humans are expected to occur in the future. Moreover, and by contrast to HeV, NiV can also be transmitted from human-to-human, with this type of transmission having contributed to more than 50% of the virus spread in Bangladesh [3,4], Philippines [5] and India [6]. The inter-human transmission extends the potential of NiV to cause deadly outbreaks in humans. Although an efficient vaccine against HeV in horses is available, neither vaccines nor therapeutic treatments are available in humans. Because of their high virulence, wide host range and interspecies transmission, HeV and NiV have been classified as biosafety level 4 (BSL4) pathogens and as potential bio-terrorism agents. NiV is even classified in the WHO blueprint list of the eight diseases for which research should be prioritized (https://www.who.int/activities/prioritizing-diseases-for-research-and-development-in-emergency-contexts) (accessed on 14 December 2021).

Henipaviruses have a non-segmented, negative-stranded RNA genome that is encapsidated by the nucleoprotein (N) within a helical nucleocapsid [7]. This nucleocapsid is the template used by the RNA-dependent RNA polymerase (RdRp) for transcription and replication. The RdRp is made of the large (L) protein and the phosphoprotein (P). The P protein is an essential polymerase cofactor as it enables recruitment of L onto the nucleocapsid template, and serves as a chaperon for both L [8,9,10] and N [11].

**Figure 1:**
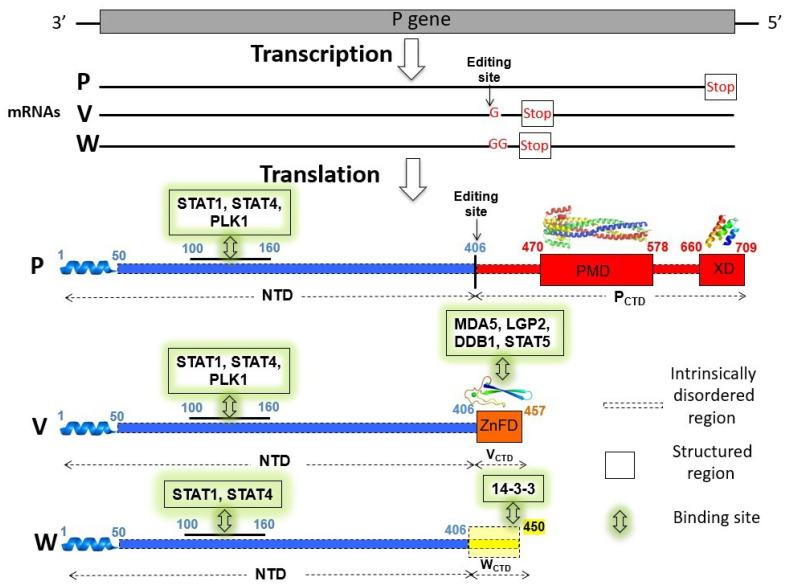
Coding capacity of the P gene and modular organization of the P, V and W proteins. The C protein is not shown for the sake of clarity. Shown is the organization of NiV proteins that is very close to that of their HeV counterpart. NTD: N-terminal region of the P, V and W proteins; P_CTD_: C-terminal region of the P protein; PMD: P multimerization domain; XD: X domain of the P protein; ZnFD: zinc-finger domain; V_CTD_ and W_CTD_: C-terminal domain of the V and W proteins. The α-helix at the N-terminus of the P, V and W proteins corresponds to the region adopting a stable α-helical conformation upon binding of P to N (i.e., N°-P_1-50_ complex) [11] or of V to host cellular transporters [44]. The structures of NiV PMD (PDB code 4N5B) [21], of a model of NiV XD [24] and of a model of the NiV ZnFD (with zinc ions shown as green spheres) [33] are displayed. The W_CTD_ is represented both as structured or disordered to reflect the lack of data on its actual conformation. Well-described interaction sites with human cell partners are shown.

The N and P proteins from henipaviruses possess long intrinsically disordered regions (IDRs) [12,13,14], i.e., regions devoid of stable secondary and tertiary structure [15,16,17,18,19]. The *Henipavirus* P protein consists of a long N-terminal intrinsically disordered domain (referred to as NTD) and a C-terminal region that encompasses both structured and disordered regions (Figure 1) [13,14,20,21,22,23,24,25].

Like in many paramyxoviruses [26], the P gene from both HeV and NiV also encodes three non-structural proteins: C, V and W. While the C protein is encoded in an alternative reading frame of the P gene, the V and W proteins (~50 kDa) result from the addition of either one (V protein) or two (W protein) non-templated guanosines at the editing site of the P messenger (Figure 1). The latter is located at the end of the region encoding the NTD of P (Figure 1). Consequently, the P, V and W proteins share a common NTD but have distinct C-terminal domains (referred to as P_CTD_, V_CTD_ and W_CTD_ respectively) (Figure 1).

The V and W proteins are key players in the evasion of the antiviral type I interferon (IFN-I)-mediated response [27,28,29]. The V protein prevents the detection of viral dsRNA by binding to melanoma differentiation-associated protein 5 (MDA5) and Laboratory of Genetics and Physiology 2 (LGP2) protein through its CTD [30], and to PLK1 (polo-like kinase) through its disordered NTD [31] (Figure 1). The V and W proteins have also an antagonist activity of IFN signaling by targeting Signal Transducers and Activators of Transcription (STAT) proteins [27]. The V and W proteins bind STAT1 through their NTD, with this ability being also conserved in NiV P [20] (Figure 1). The V protein inhibits STAT1 translocation into the nucleus and promotes STAT1 ubiquitination and degradation [32]. The V protein does so by binding to the highly conserved DNA damage-binding protein 1 (DDB1) [33] that is part of the ubiquitin ligase E3 complex. We previously showed that binding of the V protein to DDB1 requires its CTD [33] (Figure 1). The W proteins sequester STAT1 into the nucleus [34] thanks to a nuclear localization signal (NLS), consisting of the KKAR basic sequence present in their CTD (NiV W^439–442^, HeV W^437–440^) [35]. This NLS is recognized by importin α3 [36] and accounts for steady-state location of the W protein in the nucleus [37]. In addition to STAT1, the NiV P, V and W proteins also bind to STAT4, with a common region encompassing residues 114–140 with been shown to be responsible for binding to both STAT proteins [38]. Moreover, the CTD of the NiV V protein binds to STAT5 [38].

Beyond its antagonist activity of IFN signaling [35], NiV W also prevents IFN-I expression [39]. The *Henipavirus* V and W proteins also inhibit the production of chemokines in vitro and modulate the inflammatory response in vivo [40]. NiV W inhibits TLR3 signaling and activation of the TNFα- and IL-1β–induced NF-κB canonical pathway [35,39,41]. Finally, *Henipavirus* W proteins bind to 14-3-3 proteins via their CTD, with this interaction having been shown to modulate various cellular processes including apoptosis [42] and inhibition of NF-κB-induced proinflammatory response [43].

The NTD of the NiV and HeV V proteins was shown to be disordered not only in isolation [13,14,20] but also within the V protein, while their CTD adopts a zinc-finger conformation [33].

Although the *Henipavirus* W proteins have been referred to as intrinsically disordered proteins (IDPs) [29], no study has been published so far supporting their disordered nature. Albeit the disordered nature of the NTD has already been experimentally shown [13,14,20], no data are available on the W protein. The lack of regular secondary structure in the conformation adopted by the W_CTD_ when bound to importin α1 or α3 [36] or to 14-3-3 isoform σ [42] suggests that it may be disordered in isolation. Despite these hints, no experimental data have been gathered so far on the W_CTD_, nor even any bioinformatic analysis has been performed. In addition, although the NTD is disordered both in isolation and in the context of the V protein, long-range intramolecular contacts between the W_CTD_ and the NTD cannot be ruled out. Importantly, this possible crosstalk between the two moieties of the W protein might cause the NTD to adopt a specific conformation that might impart unique functions to W. For this reason, and in light of the key role that the W protein plays in counteracting the host antiviral response, investigating the conformational properties of this protein is relevant and is a prerequisite toward rational antiviral approaches. To this end, we herein have combined in silico analyses and biochemical and biophysical studies of the HeV and NiV W proteins. Our results reveal that their CTD are consistently predicted to be intrinsically disordered and we provide experimental evidence of the disordered nature of the W proteins. In addition, the W proteins were found to phase separate and to form amyloid-like fibrils.

## 2. Results

### 2.1. Bioinformatic Analysis of the HeV and NiV W Protein Sequences

The HeV and NiV W native proteins are 448 and 450 residues long, respectively. The two W sequences are close to each other (58% of identity, 71% of similarity) with their CTD displaying a high sequence identity (82%) and the region encompassing residues 270–390 exhibiting the highest divergence (Supplementary Figure S1). The isoelectric point (pI) of both proteins is acidic, while that of their CTDs is basic (Table 1). The hydropathy values of the W proteins are consistent with an overall hydrophilic nature (Table 1).

**Figure 2:**
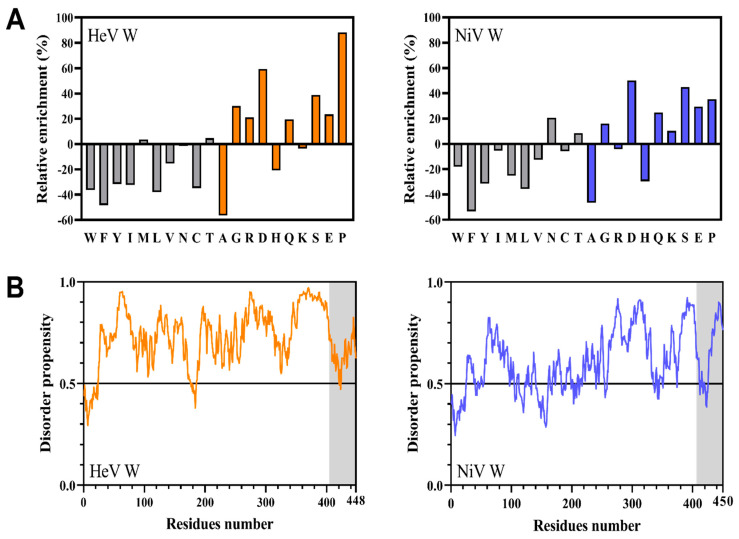
(**A**) Deviation in amino acid composition from the Swiss-PROT database of HeV and NiV W proteins. The relative enrichment in disorder promoting (orange and blue bars) and depletion in order-promoting (gray bars) residues is shown. Residues have been ordered on the x-axis according to the TOP-IDP flexibility index as described in [46]. (**B**) Disorder prediction of the W proteins as obtained by IUPred2A [48]. Residues with a disorder score above 0.5 are considered to be disordered. The region corresponding to the CTD is shaded in gray.

The sequence composition of HeV and NiV W proteins was compared to that of proteins within the SWISS-PROT database (Figure 2A). Both domains have a biased sequence composition, being enriched in disorder-promoting residues (T,A,G,R,D,H,Q,K,S,E,P), and in particular in Asp (D), Ser (S) and Pro (P), and depleted in order-promoting residues (W, F, Y, I, M, L, V, N, C) [46]. In line with these observations, both W proteins are predicted to be intrinsically disordered by the mean hydrophobicity/mean net charge ratio [47], as judged from their location in the left-hand side of the RH-plot (Supplementary Figure S2A,B).

Since their respective NTD was already reported to be intrinsically disordered [13,14,20], we were mainly interested in analyzing the sequence properties and contribution of the CTD to the overall disorder content of the proteins. To this end, we analyzed the sequence of the two proteins using IUPred2A, a disorder predictor that provides a *per* residue disorder score (Figure 2B). Both CTD are predicted to be disordered (Figure 2B, gray shaded region), although a sharp drop in the disorder score can be observed over a short stretch of residues (Figure 2B). These short regions of predicted order might correspond to MoRFs (Molecular Recognition Features), also called MoREs (Molecular Recognition Elements), i.e., short regions within IDRs with a propensity to fold upon binding to a partner or ligand [49,50].

In agreement with the IUPred2A prediction, both CTDs are predicted to be disordered by the RH-plot (Supplementary Figure S2C,D) and have a biased sequence composition similar to that of the full-length W proteins (Supplementary Figure S3A).

Since the W proteins possess four (HeV) or six (NiV) cysteines, of which three are conserved (Supplementary Figure S1), we also analyzed the sequences using the redox-dependent prediction implemented in IUPred2A. The redox state modeling option of IUPred2A enables identifying redox-sensitive regions, i.e., regions susceptible to undergo redox-dependent disorder-to-order or order-to-disorder transitions [51]. No such regions were identified (data not shown) consistent with a predicted scenario where intramolecular disulfide bridges (if any) would not trigger a disorder-to-order transition.

**Figure 3:**
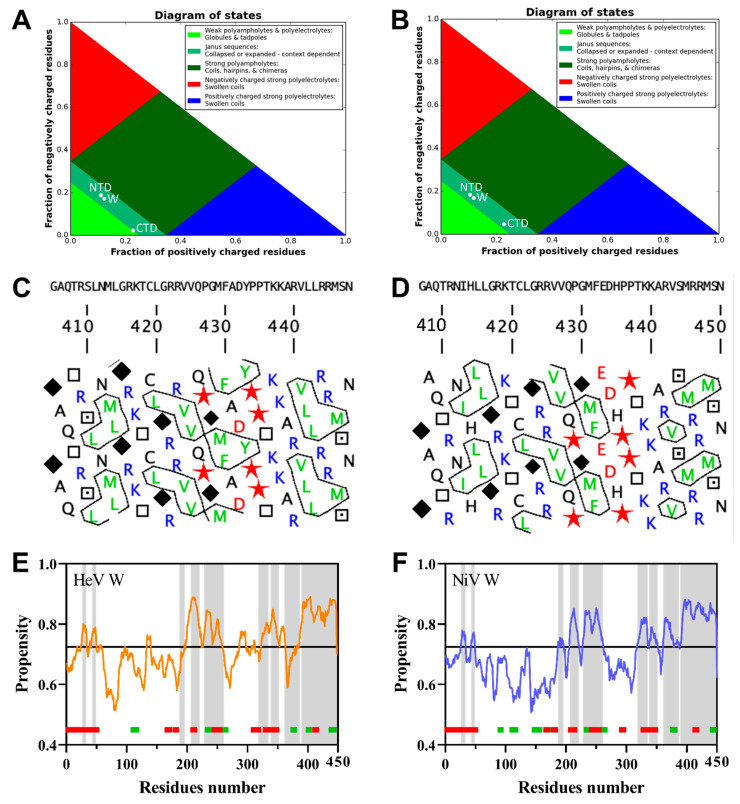
Phase diagram plots of HeV (**A**) and NiV W (**B**) proteins as provided by CIDER (http://pappulab.wustl.edu/CIDER/ (accessed on 1 April 2021) [45]. HCA plots of the CTD of HeV (**C**) and NiV (**D**) W featuring the amino acid sequence above the HCA plot. Red stars: proline residues, black diamonds: glycine residues, squares: threonine residues, dotted squares: serine residues. MoRF and secondary structure prediction within HeV (**E**) and NiV W (**F**) proteins as provided by MoRFChibi System [57] and PSIPRED [58] servers, respectively. Predicted MoRFs are shaded in gray. Predicted α-helices and β-strands are shown in red and green respectively.

Sequence polarity was shown to be a determinant of intrinsically disordered protein (IDP) compaction, with polar IDPs having been found to favor collapsed ensembles in water despite the absence of hydrophobic groups [52]. To assess sequence polarity, the net charge per residue (NCPR, defined as f_+_ − f_−_) [52,53], the total fraction of charged residues (FCR, defined as f_+_ + f_−_), and linear distribution of opposite charges (κ value) [54] were calculated for the full-length W proteins and for their respective NTDs and CTDs (Table 1). For both W proteins, as well as for their respective NTDs, the NCPR is negative, and the FCR values are close (~0.29). By contrast, the NCPR of the CTDs is positive and the FCR of NiV W_CTD_ is slightly higher (0.273) than that of HeV W_CTD_ (0.250). According to the predictive diagram of states developed by Pappu and colleagues [55], both the W proteins and their respective domains fall in phase diagram region (PDR) 2, with the CTDs being however very close to the boundary separating PDR1 and PDR2 (Figure 3A,B). PDR 2 corresponds to proteins that adopt conformations likely representing a continuum of possibilities between PDR 1, which accommodates weak polyampholytes or polyelectrolytes adopting a globular conformation, and PDR 3, which embeds strong polyampholytes adopting non-globular, swollen coil-like conformations. The κ values of the full-length proteins and of their domains are low indicating that opposite charges are well mixed and suggesting that these proteins and their domains adopt preferentially extended and swollen, coil-like conformation.

The amino acid sequences of the CTDs were also analyzed using Hydrophobic Cluster Analysis (HCA) [56] (see Appendix A). The HCA plots of the two domains show an enrichment in proline (red stars), glycine (diamonds) and basic residues (blue). Despite this bias toward disorder-promoting residues, the two CTDs are relatively enriched in hydrophobic clusters advocating for the occurrence of at least transiently populated regular secondary structure elements (Figure 3C,D). Notably, the HCA plots of both HeV and NiV W_CTD_ display a higher content in hydrophobic clusters with respect to the HCA plots of the V_CTD_ that are known to adopt a zinc-finger conformation (Supplementary Figure S3B). The latter however are enriched in cysteines that drive folding of these domains [33]. The HCA plot of HeV W_CTD_ features slightly larger hydrophobic clusters suggesting a higher extent of order compared to NiV W_CTD_ (Figure 3C,D). In line with this observation, the disorder promoting score, as provided by CIDER, is slightly larger for NiV W_CTD_ than for the corresponding domain of HeV (Table 1). In further support of a predicted higher order content in HeV W_CTD_, PSIPRED analysis predicts an additional β-strand in this domain (Figure 3E,F).

The pattern of predicted secondary structure elements in HeV W_NTD_ is very similar to the one of the cognate protein, as is the pattern of predicted MoRFs (Figure 3E,F). Predicted MoRFs overlap with predicted secondary structure elements reflecting possible partially pre-configured MoRFs. The largest predicted secondary structure element is an α-helix encompassing the first 50 residues and corresponding to the region adopting a helix-kink-helix conformation in the crystal structure of the N°-P_1-50_ complex of NiV [11]. The other predicted MoRFs are in good agreement with experimentally observed secondary structure elements as revealed in NiV NTD by NMR spectroscopy [14,20] as well as with already mapped binding sites, including the STAT1 (aa 110–140) [20], STAT2 (aa 110–140 and 230–237) [59] and STAT4 (aa 110–140) [38] binding sites. Interestingly, the CTD of both W proteins is entirely predicted to be a MoRF (Figure 3E,F), reflecting its inherent propensity to fold upon binding and consistent with its expectedly broad molecular partnership as judged from the wide panel of functions specific to the W protein.

Analysis of the amino acid sequence of the CTD of the two W proteins with the Phyre2 protein fold recognition server [60] returned a high confidence (99.6%) model based on the crystal structure of the HeV W_CTD_ in complex with importin α1 or α3 [36]. The model encompasses 59% of the CTD residues and consists of an irregular conformation (data not shown).

Altogether, results support the conclusion that W_CTD_ is an IDR with a high propensity to fold upon binding.

### 2.2. Expression and Purification of the W Proteins

Because the cognate V proteins devoid of any solubility tag were found to be expressed in inclusion bodies [33], we decided to subclone the W genes into the pETG-20A vector that allows the expression of a cleavable hexahistidine tagged TRX fusion protein. Although the fusion proteins were found to be soluble, attempts at purifying them by IMAC revealed that the majority of the protein did not bind to the resin (data not shown). Moreover, TEV protease failed to cleave off the TRX-His_6_ tag (data not shown). We ascribed both events to poor accessibility of the His_6_ and TEV cleavage site and resorted to clone the W gene into the pDEST17OI vector that drives the inducible expression of a hexahistidine tagged form of the protein with no solubility tag. As in the case of the V proteins [33], the W proteins expressed from this vector were found to be expressed in inclusion bodies only, irrespective of the IPTG concentration, temperature of induction, and culture medium used (data not shown).

**Figure 4:**
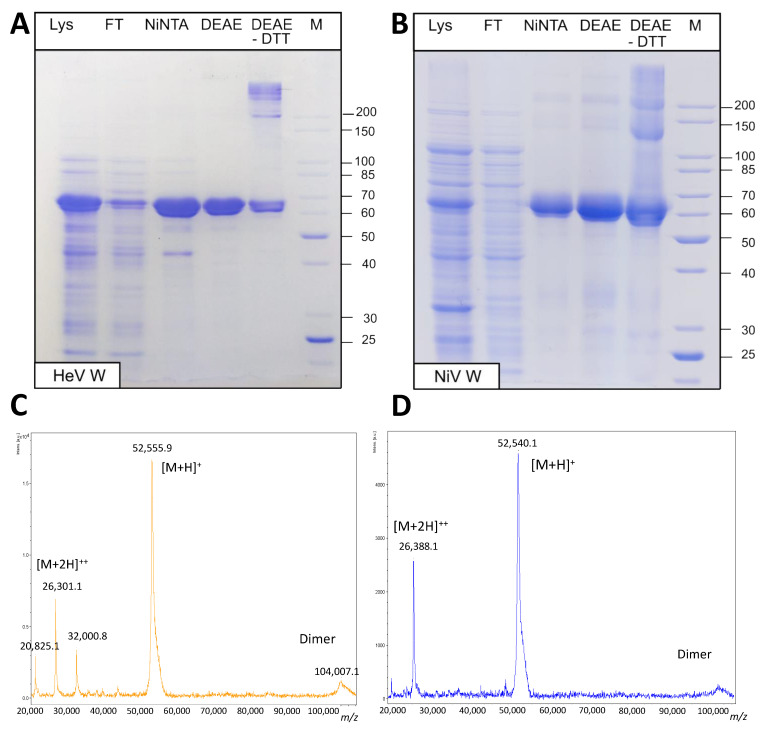
(**A,B**) Coomassie blue staining of a 15% SDS-PAGE analysis of the purification of the HeV (**A**) and NiV (**B**) W proteins. Lys: clarified lysate (soluble *plus* insoluble fraction); FT: flow-through of IMAC; NiNTA: eluent from IMAC; DEAE: eluent from IEC; DEAE−DTT: eluent from IEC under non-reducing conditions; M: molecular mass markers (in kDa). (**C**,**D**) MALDI-TOF-TOF mass analysis of HeV (**C**) and NiV (**D**) W proteins. The HeV W protein generated an average mass of 52,577.55 Da for a theoretical mass of 52,573.89 Da (without the initial methionine) (**C**). The NiV W protein generated an average mass of 52,656.8 Da for a theoretical mass of 52,744.15 Da (without the initial methionine) (**D**).

The W proteins were thus purified from inclusion bodies under denaturing conditions in two steps: IMAC and IEC. As shown in Figure 4A,B the final purified W proteins migrate with an apparent molecular mass of ~65 kDa, a value 1.2 times higher than the one expected from the amino acid sequence (~53 kDa). Aberrant electrophoretic migration, which had been already reported for *Henipavirus* NTD [13] and V [33], is a hallmark of IDPs and is mainly due to their typical compositional bias, e.g., high net charge and low hydrophobicity that causes them to bind less SDS than globular proteins [61,62]. In the case of the W proteins, this anomalous electrophoretic behavior could also be at least partly due to their high proline content (8.4% in HeV W and 6.1% in NiV W to be compared to 4.7% for proteins in the SWISS-PROT database) [62,63].

For both proteins, mass spectrometry (MS) analysis of peptides resulting from tryptic digestion confirmed the identity of the purified proteins (Supplementary Figure S4). For both proteins, the profile resulting from tryptic digestion of the protein (Supplementary Figure S4) is consistent with the initial methionine having been cleaved off, an event predicted to occur with an 84% frequency for proteins in which the second residue is a serine [64]. MALDI-TOF MS analysis of the HeV W protein revealed the presence of a species with an average molecular mass of 52,577.55 Da, a value very close (i.e., 3.66 Da of difference) to the expected value calculated from the amino acid sequence without the initial methionine (Figure 4C). In the case of NiV, the experimentally observed average molecular mass (52,656.8 Da) is a bit more divergent (87.54 Da) but still close to the value expected for a form in which the initial methionine has been cleaved off (Figure 4D). Furthermore, for both proteins an additional, much less intense peak with a mass close to that expected for a dimeric form was observed (Figure 4C,D). In further support of the ability of the recombinant proteins to form disulfide-bridged oligomeric species, SDS-PAGE analysis in non-reducing conditions revealed the presence of higher mass species that are no longer detectable upon addition of DTT (Figure 4A,B).

### 2.3. Protease Sensitivity of the W Proteins

**Figure 5:**
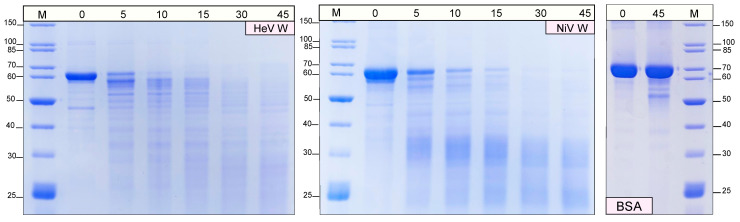
SDS-PAGE analysis of a limited thermolysin digestion of HeV and NiV W proteins and of BSA (control) at various time intervals (minutes). M: molecular mass makers (in kDa).

High sensitiveness to proteolysis is a hallmark of structural disorder (see [62,65] and references therein cited). The use of thermolysin, which has a broad substrate specificity, allows the identification of cleavage sites solely on the basis of the flexibility of the protein substrate. To assess the extent of protease sensitivity, the W proteins were submitted to digestion by thermolysin. As shown in Figure 5, both proteins start to be degraded after 5 min of incubation and are extensively degraded after 45 min, a behavior that is consistent with the lack of a packed core and with an overall solvent accessibility. Conversely, BSA was shown to be resistant to proteolysis even after an incubation of 45 min (Figure 5). Interestingly, the two proteins do not exhbit the same extent of susceptibility to proteolysis. Indeed, while a 45-min digestion of the NiV W protein results in an enrichment in small (i.e., <35 kDa) molecular mass fragments, in the case of HeV W after 45 min of incubation protein fragments of higher molecular mass (in the 60–25 kDa range), are still discernible (Figure 5).

### 2.4. Differential Scanning Fluorimetry of the W Proteins

**Figure 6:**
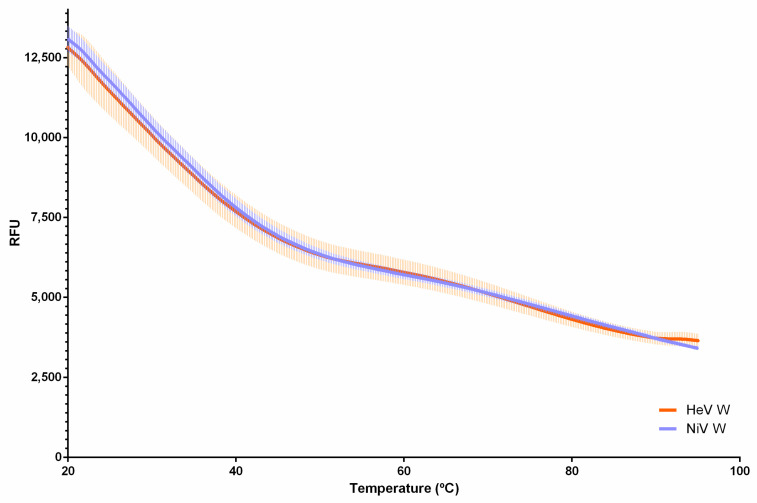
Differential scanning fluorimetry (DSF) of HeV and NiV W proteins in the presence of Sypro Orange in the 20–95 °C temperature range. The results are the mean of 9 replicates. Error bars correspond to the standard deviations.

We further analyzed the W proteins by differential scanning fluorimetry (DSF). DSF is a broadly use technique to determine the conformational stability of proteins; it relies on the use of a fluorescent dye whose fluorescence is enhanced when protein hydrophobic cavities become accessible as a result of temperature-induced protein unfolding. IDPs, which do not possess hydrophobic cavities being devoid of a stable 3D structure, are characterized by a fluorescence profile that lacks the typical transition peak observed for structured proteins and is thus rather flat and temperature-independent (see [62,65] and references therein cited). The experimentally observed profile obtained for both the W proteins does not feature any transition peak, consistent with the lack of a stable 3D structure (Figure 6). These results provide additional support for an overall disordered nature of the W proteins, in line with the in silico analyses and with the above-described experimental lines of evidence.

### 2.5. Hydrodynamiques Properties of the W Proteins from Size Exclusion Chromatography (SEC)

**Figure 7:**
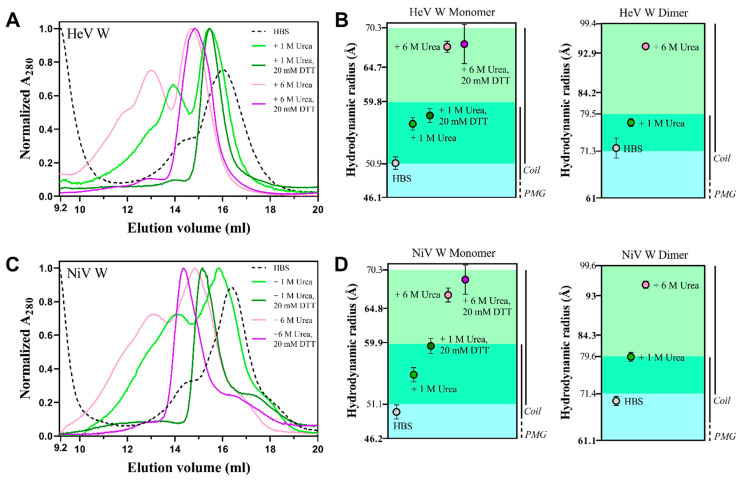
(**A**,**C**) Normalized SEC elution profiles of HeV (**A**) and NiV (**C**) W, as obtained using various conditions. Shown are the results of one out of three independent experiments. (**B**,**D**) Stokes radii (R_S_) of the monomeric and dimeric form of HeV (**B**) and NiV (**D**) W in the different conditions. The regions corresponding to PMG (light blue) and Coil (green) forms are highlighted. Values shown in bold in panels B and D correspond to the average values expected for PMG (smallest values), Coils (intermediate values) and Denatured (largest values) conformations. The errors bars correspond to the experimentally observed s.d. in three independent experiments.

To infer the Stokes radii (R_s_) of the HeV and NiV W proteins we used analytical SEC. The elution profile of both proteins in HBS at pH 7 features a major peak at ~16 mL preceded by a shoulder at ~14 mL. However, for both proteins the highest peak is at ~9 mL, indicating that the protein mostly exists in an aggregated form in solution (Figure 7A,C). SDS-PAGE analysis under reducing and non-reducing conditions showed that the species eluted at ~14 mL corresponds to a dimeric form of the protein stabilized by disulfide bridges (data not shown). These results are in line with the MS data that pointed out the presence of a less abundant dimeric species under non-reducing conditions. When we attempted at eliminating the dimeric species by adding DTT, we noticed that both proteins precipitated unless urea, at a concentration of at least 1 M, was added. SEC under reducing conditions was therefore performed by supplementing the buffer with urea.

We first carried out SEC in the presence of 1 M urea to investigate possible effects on the conformational properties of the proteins. The addition of 1 M urea triggers a slight (~0.5 mL), though significant, shift in the elution volume of the peak corresponding to the monomeric form of the proteins (Figure 7A,C), reflecting a ~5.5 Å increase in the derived R_S_ (Figure 7B,D). Beyond this effect, the addition of 1 M urea also impacts the overall profile. For both proteins, upon addition of 1 M urea, a pronounced peak at ~14 mL is observed reflecting an increased amount of the dimeric form (Figure 7A,C). The latter is paralleled by the disappearance of the major peak at ~9 mL and by the concomitant accumulation of higher mass species eluted between 10 and 14 mL (Figure 7A,C). As expected, addition of 6 M urea results in an even more pronounced change in the elution volume of the monomeric species that is further reduced by ~0.9 mL (Figure 7A,C). This shift in the elution volume entails a change in the R_S_ that is increased, with respect to the form obtained in native conditions, by ~16.6 Å (Figure 7B,D). For both proteins, a very similar trend for the impact of urea, i.e., a progressive reduction in the elution volume and an increase in the R_S_, was also observed for the peak of the dimeric form (Figure 7A,B). The sensitivity of the W proteins to urea likely reflects the presence of residual secondary and/or tertiary structure typical of the premolten globule (PMG) state [66].

As expected, for both proteins, the addition of 20 mM DTT triggers the disappearance not only of the peaks corresponding to the dimeric species but also of those corresponding to the higher mass species (Figure 7A,C). Notably, for both proteins, the addition of DTT induces a shift in the elution volume of the peak of the monomeric species (Figure 7A,C), therefore leading to an increase in the corresponding R_S_ (Figure 7B,D). While in the case of HeV the increase is barely significant, it is more pronounced in the case of NiV (Figure 7B,D). This behavior indicates that the cysteine redox state impacts the compaction of the protein, consistent with a scenario where one (or more) intramolecular disulfide bridges would contribute to compact the chain, and disulfide bridge reduction would lead to expansion of the polypeptide chain. The NiV W protein has 6 cysteines, while the HeV W has only 4. As such, the NiV W has a higher disulfide bridge-mediated compaction potential compared to HeV W, which may explain the more pronounced effect of DTT on NiV W compared to HeV W.

From the elution volume of the major species (16.0 ± 0.08 mL for HeV W and 16.1 ± 0.07 mL for NiV W), as obtained using HBS as elution buffer, the corresponding R_S_ was estimated to be 51.0 ± 0.9 Å for HeV W and 50.1 ± 0.8 Å for NiV W (Table 2). By comparing the mean measured Stokes radius (R_s_^obs^) for HeV and NiV W with the theoretical Stokes radii expected for various conformational states (R_s_^NF^: natively folded protein; R_S_^PMG^: expected for a PMG; Rs^U^: fully unfolded form; R_S_^IDP^: expected for an IDP), the two proteins were found to have R_S_ values ~1.7 times larger than the value expected for a natively folded protein, and close to the one expected for a PMG (Table 2). Similarly, the R_S_ of the dimeric form of the W proteins was estimated to be ~72 Å, a value suggesting a disulfide-bridged dimer in a PMG conformation (Figure 7B,D).

These results, while providing additional experimental evidence for the prevalently disordered nature of both W proteins, indicate the presence of some transiently populated secondary and/or tertiary structure typical of the PMG state, also supported by the expansion effect observed in the presence of urea. The compaction index of the W proteins indicates that NiV W is slightly more compact than HeV W (Table 2), in agreement with bioinformatic analyses that overall predict NiV W to be slightly less disordered than HeV W (Figure 2B and Table 1).

### 2.6. Far-UV Circular Dichroism (CD) Studies of the W Proteins

**Figure 8:**
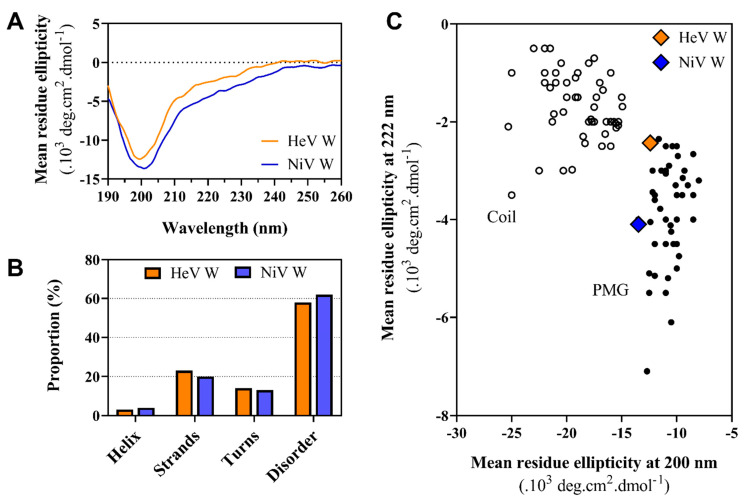
(**A**) Far-UV CD spectra of HeV and NiV W at 1 μM in 10 mM sodium phosphate pH 7. All spectra were recorded at 20 °C. Data are representative of one out of three independent measurements. (**B**) Secondary structure content of the two proteins, as derived using Dichroweb (CDSSTR algorithm, set 7). (**C**) Plot of the molar residue ellipticity (MRE) at 222 nm and at 200 nm of a set of well-characterized unfolded, random coil-like (Coil, empty circles) or PMG-like (PMG, full circles) proteins (from [66]). The position in the plot of HeV W and NiV W is highlighted.

To evaluate the secondary structure content of the HeV and NiV W proteins, circular dichroism (CD) measurements were performed in the far ultraviolet (UV) region. Under native conditions and neutral pH, both spectra display a large negative peak centered at 200 nm, low intensity in the 220–230 nm region, and low ellipticity at 190 nm (Figure 8A). The spectra are typical of disordered proteins lacking any stable organized secondary structure. For both proteins, spectral deconvolution revealed a high content (about 60%) of unordered structure along with a ~20% content in β-strands (Figure 8B).

Far-UV CD spectroscopy enables discriminating between IDPs adopting a random coil-like (RC) state and a PMG-like state, based on the ratio of the ellipticity values at 200 and 222 nm [66]. According to their ellipticity values at 200 and 222 nm, both W proteins fall in the PMG-like region of the plot (Figure 8C). These results confirm the hints obtained from SEC experiments and support the classification of the W proteins within the PMG-like subfamily of IDPs.

We next sought at assessing whether disulfide bridges could play a role in stabilizing transiently populated regular secondary structure elements within the W proteins. Since both proteins precipitate in the presence of DTT unless 1 M urea was added, we recorded the CD spectra of the two proteins in the presence of 1 M urea supplemented with 10 mM DTT, and in the presence of solely 1 M urea as a control (Supplementary Figure S5A,B). While the spectrum of NiV W in the presence of 1 M urea is perfectly superimposable onto that obtained in the absence of urea (Supplementary Figure S5B), the spectrum of HeV W slightly deviates from that recorded in sodium phosphate buffer without urea (Supplementary Figure S5A), indicating that at this concentration urea has a poor (HeV) or no (NiV) impact on the secondary structure content. In light of this, the expansion effect triggered by 1 M urea as observed in SEC studies, can be accounted for by uniquely a destabilization of the transiently populated tertiary structure.

Although the spectra of both proteins in the presence of urea and DTT are quite noisy, a significant deviation from the respective control spectra is discernible (Supplementary Figure S5A,B). In particular, a decrease in the ellipticity at 220 and 215 nm is observed, reflecting respectively a reduction in the α and β content (Supplementary Figure S5A,B). These results advocate for a role of disulfide bridges in the stabilization of secondary structure elements and mirror those obtained by analytical SEC pointing to a role of oxidized cysteines in driving protein compaction (Figure 7).

One of the functional advantages of IDPs arises from their ability to contextually change their conformation due to binding or to environmental changes. To assess the folding potential of the W proteins, their far-UV CD spectra were recorded in the presence of TFE. This organic solvent is a secondary structure stabilizer that is used to mimic the hydrophobic environment experienced by proteins during interactions [67,68]. It is thus widely used as a probe of hidden structural propensities to unveil protein regions with a propensity to undergo induced folding [62,65].

**Figure 9:**
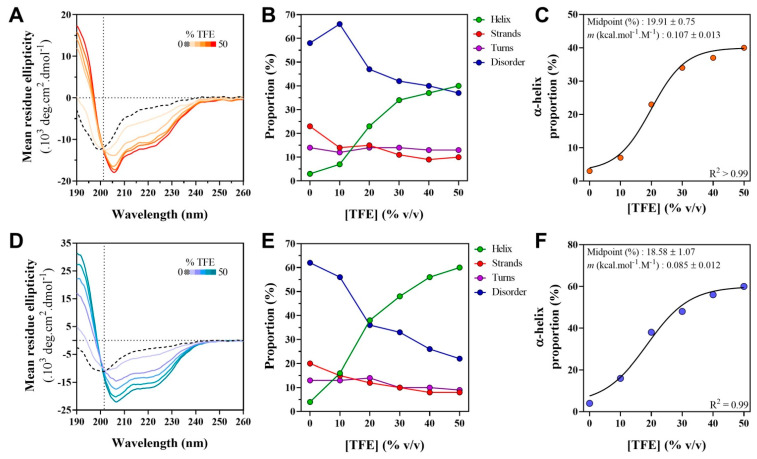
(**A**,**D**) Far-UV CD spectra of HeV (**A**) and NiV (**D**) W proteins in the presence of increasing concentrations of TFE at 20 °C. Proteins were at 1 μM in 10 mM sodium phosphate pH 7. Data are representative of one out of two independent experiments. The vertical line is centered on the isodichroic point. (**B**,**E**) Secondary structure content of HeV (**B**) and NiV (**E**) W proteins, as derived using CDSSTR, as a function of TFE concentration. (**C**,**F**) Transition diagrams of HeV (**C**) and NiV (**F**) W proteins. The midpoint of transition and *m* values, as derived from the fitting, along with the quality of the fitting (R^2^) are shown.

For both proteins, addition of TFE induces a gain of α-helicity indicated by the characteristic maximum at 190 nm and double minima at 208 and 222 nm (Figure 9A,D). The estimated α-helical content gradually increases upon increasing the TFE concentration from 0 to 50% (Figure 9B,E). For the two proteins, the spectra display an isodichroic point at 201 nm indicative of a two-state transition (Figure 9A,D). We therefore plotted the percentage of α-helix as a function of the TFE concentration and fitted the data to a sigmoidal curve, corresponding to a two-state transition (Figure 9C,F). The fitting yielded a midpoint of transition at a TFE concentration of ~20% for both proteins. From the fitting procedure, the *m* value could also be obtained. The latter is a measure of the osmolyte efficacy in folding or unfolding a protein. The *m* value is typically positive for protecting osmolytes, such as TFE that drive the equilibrium toward the folded state. In agreement, *m* values of 0.107 ± 0.013 kcal mol^−1^ M^−1^ and of 0.085 ± 0.012 kcal mol^−1^ M^−1^ were obtained for HeV W and NiV W, respectively. The two proteins thus have midpoints and *m* values very close to each other, indicating that they follow a very similar folding pathway. Despite this similarity however, the α-helical propensity of the NiV W protein is more pronounced, as judged from the higher α-helical content of the latter with respect to the cognate protein at each point of the TFE titration.

### 2.7. Small-Angle X-ray Scattering (SAXS) Studies of the W Proteins

To achieve a more quantitative description of the conformational properties of the W proteins in solution, we performed SAXS studies coupled to SEC (SEC-SAXS). To this end, we first recorded SEC-SAXS data under native and non-reducing conditions (i.e., in the absence of urea and DTT) with the hope that the SEC step would enable analysis of the monomeric species on its own. Unfortunately, however, obtained data turned out to suffer from polydispersity and to be unexploitable (data not shown). To eliminate disulfide-bridged oligomeric species, we resorted recording data under reducing conditions. Because DTT causes the proteins to precipitate unless 1 M urea is added, and knowing that the addition of urea dissolves the higher molecular mass aggregates detected by SEC but also visible in solution, we performed SEC-SAXS experiments in the presence of both 1 M urea and 5 mM DTT while being aware that these conditions trigger a slight extension of the chain, as judged from analytical SEC experiments. The rationale for adding 5 mM DTT rather than 20 mM as in analytical SEC experiments, resides in the fact that this DTT concentration was shown to be sufficient to reduce the protein (data not shown) and that we wanted to minimize the concentration of buffer additives so as to achieve maximal contrast between the protein and the solvent.

**Figure 10:**
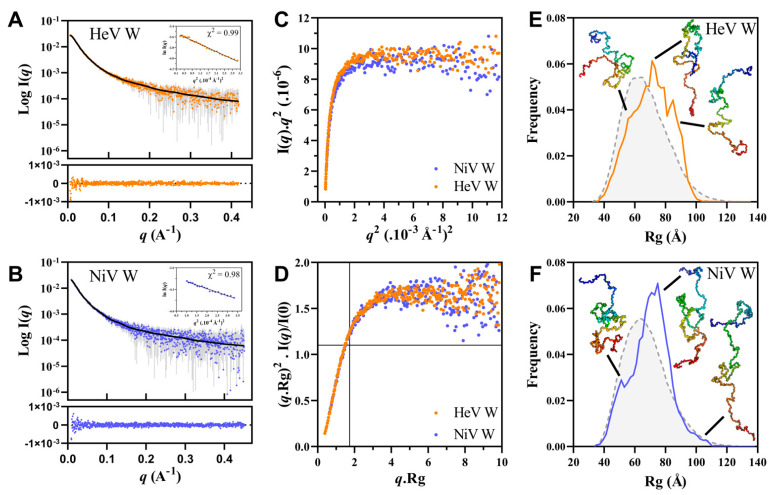
(**A**,**B**) Scattering curves of HeV (**A**) and NiV (**B**) W proteins and ensemble EOM 2.0 fits (solid line). Below are shown the plots of residuals. Insets: Guinier plots with the quality of the fit (χ^2^). Scaled Kratky-Debye (**C**) and normalized Kratky (**D**) plots of the scattering data of HeV (orange) and NiV (blue) W. (**E**,**F**) R_g_ distributions of the initial pool generated by EOM 2.0 (gray) and of the corresponding selected ensemble for the HeV (**E**) and NiV (**F**) W proteins. A cartoon representation of three conformers present in the ensembles is also displayed. The structures were drawn using Pymol 2.0.1 [71].

Despite the presence of minor peaks corresponding to aggregates, for both W proteins the obtained SEC profiles featured a sharp and symmetric peak corresponding to the monomeric form (Supplementary Figure S6A,B). In both cases, Guinier analysis of the deconvoluted scattering curves (Figure 10A,B) revealed linearity in the Guinier region (i.e., for qR_g_ < 1.3), with no indication of protein aggregation (Figure 10A,B insets). The R_g_ extracted from Guinier analysis is 72.01 ± 0.89 Å for HeV and 70.96 ± 1.27 Å for NiV W. The R_g_ values derived from Guinier anaysis are close to those derived from the P(r) (73.62 ± 0.40 Å for HeV and 73.62 ± 0.62 for NiV W). The theoretical R_g_ values expected for IDPs of the same length, calculated using Flory’s power law (see Equation (9) in Materials and Methods), are 63.5 Å for HeV and 63.6 Å for NiV. The experimentally determined R_g_ values are therefore larger than those expected for IDPs possessing the same number of residues and close to value expected for chemically denatured proteins (~78 Å). This finding is in line with what could be expected given the use of a buffer supplemented with 1 M urea and 5 mM DTT, which jointly were found to trigger a ~13% expansion of the protein chain (see Figure 7). The R_g_-based compaction index (CI) (see Equation (10) in Materials and Methods) of NiV W is 0.111, a value slightly, though significantly, larger than that of the cognate HeV W protein (0.089). This finding mirrors differences observed in R_S_-based CI as estimated from SEC (see Table 2).

The pairiwse distance distribution, P(r), yielded a maximal dimension, D_max_, of ~240 Å for HeV and ~245 Å for NiV W (Supplementary Figure S6C) with a long tail in the P(r) function, indicating that the proteins assume an overall non-compact conformation [69]. The ratio between the experimentally observed R_g_ and R_S_ is ~1.41 for both HeV and NiV W proteins. This value is closer to the one expected for RC-like IDPs (1.5) than to that expected for PMG-like IDPs (1.0) [70], probably due to the expansion effect of urea and DTT. The overall SAXS parameters for the W proteins are listed in Table 3.

The flexible nature of the W proteins was also qualitatively assessed using the Kratky-Debye and the normalized Kratky plots (Figure 10C,D). The presence of a plateau in the Kratky-Debye plot (Figure 10C) and the shape of the normalized Kratky plot (with no clear maximum) (Figure 10D) indicate that the W proteins are intrinsically disordered.

To achieve further insights into the conformational behavior of the W proteins, we used SAXS data in search of an ensemble that quantitatively describes the conformational behavior of the proteins in solution. To this end, we used the program EOM 2.0 (see Material & Method section). For both proteins, the scattering curve back-calculated from the selected ensemble (Figure 10A,B solid lines) fits well the experimental SAXS data as judged from both χ2 and CorMap [72] (χ2 = 0.52, *p* = 0.53 for HeV W and χ2 = 0.44, *p* = 0.014 for NiV W). For both proteins, the resulting final R_g_ (Figure 10E,F) and Dmax (Supplementary Figure S6D) distributions, are unimodal and close to those of the initial pool, indicating that the W proteins exist in solution as randomly distributed ensembles of non-compact and highly flexible conformations. Please note that successive and independent selections by EOM 2.0 consistently yielded similar R_g_ distributions thus attesting the reproducibility of the results (data not shown). The flexibility of the ensemble was quantified through the Rflex that was estimated to be ~82.5% for HeV W and ~82% for NiV W (to be compared to ~84.7% and to 84.5% for the intial HeV and NiV W pool, respectively).

The average R_g_ value of the two ensembles (71.7 Å for HeV and 70.7 Å for NiV) is slightly larger than that of the initial random pool, consistent with the expanding effect of the combined use of urea and DTT.

In conjunction with all the other experimental lines of evidence presented above, SAXS data definitely demonstrate that the W proteins are intrinsically disordered.

### 2.8. Phase Separation and Fibrillation Abilities of the W Proteins

IDPs and/or IDRs are known for their involvement in a broad range of phase separation behaviors [73,74,75]. IDRs can drive liquid-liquid phase separation (LLPS) and the resulting biomolecular condensates can undergo “maturation” toward a gel or solid state [74,76,77,78]. IDRs are also known to form amyloid-like structures that can form either from liquid samples or from hydrogels [79,80,81]. In line with these properties, we previously reported that HeV V jellifies in vitro. The minimal HeV V region responsible for this ability (referred to as PNT3, aa 200–310 of HeV V) was identified within its intrinsically disordered NTD [82,83]. Binding assays to an amyloid-specific dye and negative-staining transmission electron microscopy (TEM) studies, showed that PNT3 forms amyloid-like fibrils [82,83].

**Figure 11:**
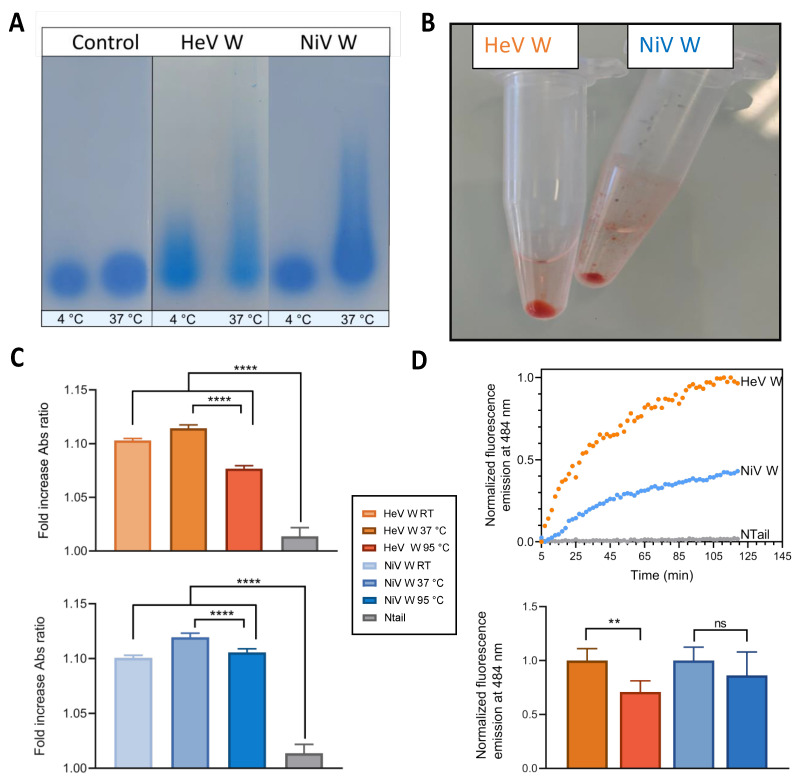
(**A**) SDD-AGE analysis of HeV and NiV W proteins, as well as of an irrelevant control protein (conalbumin, able to form amorphous aggregates upon heating), following an incubation of 48 h at either 4 °C or 37 °C. (**B**) CR binding by purified HeV & NiV W proteins. Proteins were incubated at 75 μM in the presence of 18 μM CR for three days at room temperature. A dense phase on the bottom of the tube that binds CR can be observed. (**C**) Fold increase in the ratio between the absorbance at 515 and at 497 nm, with respect to a sample containing CR alone, of a HeV W or NiV W or N-tail sample at 20 μM after 27 h of incubation at RT or at 37 °C. Following incubation at 37 °C, the W proteins were also heated at 95 °C for five minutes. N-tail was chosen as a control IDP devoid of aggregation and fibrillation propensities. The error bar corresponds to the standard deviation, with n = 3. The four asterisks denote a statistically significant difference (*p* < 0.0002) with respect to the control (One-way ANOVA test). (**D**) Upper panel: Thioflavin T (ThT) binding assays of the W proteins and of a control protein (N-tail) at various time points of incubation at 37 °C. Proteins were incubated at 40 μM in the presence of 40 μM ThT. Data are representative of two independent experiments. Lower panel: Normalized fluorescence as obtained after 2 h of incubation at 37 °C followed by an additional incubation step of five minutes at either 37 °C or 95 °C. The error bar corresponds to the standard deviation, with n = 3. The two asterisks denote a statistically significant difference (*p* < 0.0069) with respect to the control (One-way ANOVA test). ns: not significant.

Because V and W possess both the minimal region conferring phase separation and fibrillation abilities (i.e., PNT3), we reasoned that W might phase separate and form fibrils as well. To ascertain this ability, we first analyzed the W proteins using Semi-Denaturing Detergent-Agarose Gel Electrophoresis (SDD-AGE). This technique enables distinguishing between amyloid-like protein polymers, which are very stable and withstand treatment with 2% SDS, and aggregates, which are bundles of polymers that dissociate under these conditions [84]. For both W proteins, SDD-AGE showed the presence of SDS-resistant oligomers, whose abundance increases by increasing the temperature of incubation from 4 °C to 37 °C (Figure 11A). By contrast, no such oligomers were observed for a sample of conalbumin preincubated at 65 °C for 30 min (Figure 11A), a condition known to lead to amorphous aggregates [85]. The HeV W protein was found to have a higher propensity to form amyloid-like fibrils as judged from the fact that at 4 °C SDS-resistant aggregates are discernible in the case of HeV but not for NiV (Figure 11A).

Both W proteins were found to phase separate and to form macroscopically observable condensates that sediment at the bottom of Eppendorf tubes, and that bind Congo Red (CR), an amyloid-specific dye (Figure 11B). The CR binding condensate was found to be slightly bigger in the case of the HeV W protein. To quantify this phenomenon, we spectrophotometrically measured the red shift of the absorbance maximum in the CR spectrum of a sample containing either the HeV W or NiV W protein. CR binding to amyloids (i.e., cross β-sheet enriched structures), is known to lead to hyperchromicity and to a red shift. The addition of either HeV W or NiV W, but not of an irrelevant, control IDP with no propensity to fibrillate (i.e., N-Tail), does indeed promote a shift in the CR spectrum from 497 nm to 515 nm (Figure 11C). To assess the stability of the amyloid-like fibrils, the W samples were also heated at 95 °C for five minutes prior to spectrophotometric measurements. Although for both proteins this heating step was found to lead to a significant decrease in the red shift, with this effect being more pronounced in the case of HeV, it does not abolish the ability of the proteins to bind CR, advocating for an at least partial heat stability of the fibrils (Figure 11C).

To further confirm the ability of the W proteins to form amyloid-like fibrils, we carried out binding assays with Thioflavin T (ThT), another well-known amyloid-specific dye [86]. Binding of ThT to the W proteins, but not to the control N-Tail protein, was found to induce an enhancement in the intensity of the ThT fluorescence emission spectrum in a time-dependent manner, although the extent and kinetics of the fluorescence increase is not the same for the two proteins (Figure 11D, upper panel).

In particular, the kinetics of fluorescence enhancement of the NiV W protein is slower and reaches a lower value (~one half of the value reached by the HeV W protein) (Figure 11D, upper panel). Following two hours of incubation at 37 °C, an additional incubation step of the W samples at 95 °C for five minutes was found to trigger a significant decrease in the fluorescence only in the case of HeV (Figure 11D, lower panel). In line with the CR experiments described above, heating does not fully abrogate ThT binding thus supporting ability of the fibrils to partly resist to heat treatment. The lack of significant impact of the heating step on the fluorescence of the NiV W protein confirms the higher heat resistance of the fibrils made of NiV W compared to those made of HeV W.

**Figure 12:**
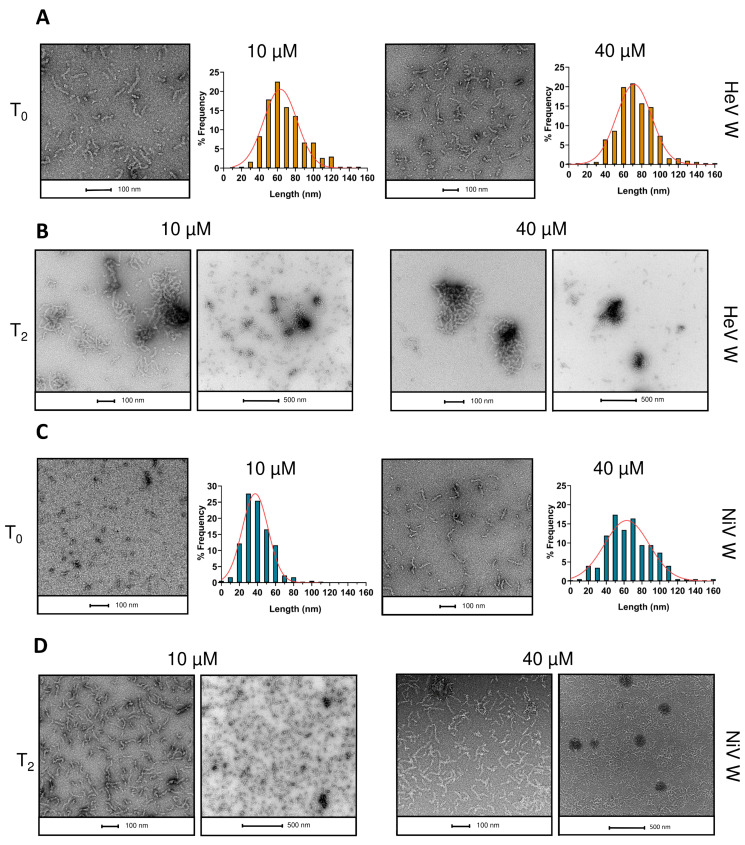
Negative-staining TEM analysis of HeV and NiV W proteins. (**A**) t0 (0 h) of incubation at 37 °C and relative fibril length distribution for the HeV W protein at 10 µM (left side) or 40 µM (right side). (**B**) t2 (10 h) of incubation at 37 °C for the HeV W protein at 10 µM or 40 µM. (**C**) t0 (0 h) of incubation at 37 °C and relative fibril length distribution for the NiV W protein at 10 µM (left side) or 40 µM (right side). (**D**) t2 (10 h) of incubation at 37 °C for the NiV W protein at 10 µM or 40 µM. Note that in all cases, samples were diluted to 1.6 µM prior to being deposited on the grid. The distribution of fibril length, as obtained from analysis of the end-to-end distance of 250–300 fibrils for each data set at time zero, is shown. Each bar in the histogram, centered on *n*, corresponds to fibrils whose length is comprised between *n* − 4 and *n* + 5. The analysis was done using the ImageJ software.

Finally, we analyzed the purified W proteins using negative-staining TEM, which unambiguously revealed the presence of fibrils for both HeV and NiV W (Figure 12). The formation of amyloid-like fibrils by the W proteins was monitored on samples at different concentrations (10 and 40 µM) and as a function of time. In the case of the HeV W protein, the two samples at the two concentrations show a very similar evolution over time (Figure 12A,B): at t0, short fibrils are visible in both grids, suggesting an almost instantaneous formation after the removal of the denaturing agent. Statistical analysis of fibril length (end-to-end distance) at t0 shows a highly similar Gaussian distribution for the two samples, centered on 60 nm for the sample at 10 µM, and at 70 nm for the sample at 40 µM, indicating that at the higher concentration longer fibrils are formed (Figure 12A). At t2 (10 h of incubation) the protein solution at 40 µM is turbid, and solid floccules are visible to the naked eye (Supplementary Figure S7A). This observation is mirrored in Figure 12B, where for both concentrations three-dimensional fibril clusters are visible. However, the size of the agglomerates observed at t1 (2 h of incubation) is about 3 times larger than that observed at t2 (Supplementary Figure S7B). This can be explained by the macroscopic aggregation observed in solution, suggesting that aggregates formed at t2 from the 40 µM sample are so large to experience fragmentation and breakage during the process of dilution and transfer on the grid. In fact, while in the 10 µM sample agglomerates of variable size and single fibrils are still visible, at 40 µM small agglomerates are present, but single fibrils and intermediate stages of aggregation are absent (Figure 12).

In the case of the NiV W protein, although the final protein concentration in the grids is the same (1.6 µM), the difference between the two concentrations at t0 is more pronounced, both in terms of fibril length and abundance, probably due to slower formation kinetics (Figure 12C). At t0 a high percentage of protein is still in the monomeric state, undetectable on the grid. In addition, for the sample at 10 µM, small, elongated structures of a few nm are present, but disappear upon incubation for 10 h (Figure 12D). Those structures are also discernible, although less abundant, in the sample at 40 µM (Figure 12C). As in the case of the less concentrated sample, they disappear upon incubation for either 2 (Supplementary Figure S7C) or 10 h (Figure 12D). This suggests that they might be germinating seeds of fibrils, where elongation takes place. Comparing the two NiV W Gaussians at t0, one can see an increase in the length of the fibrils with increasing concentration (distribution centered on 40 nm for the 10 µM sample, 60 nm for the 40 µM sample). Upon increasing the incubation time to 10 h (t2), a moderate elongation of the fibrils can be observed for both concentrations, leading to a homogeneous layer on the grid. In both cases, three-dimensional assembly structures between the fibrils are absent, in contrast to HeV W.

For both proteins, fibril width was also measured (Supplementary Figure S7D), and was found to remain constant over time and to be independent from the initial sample concentration (data not shown). The obtained distributions, averaged over all the incubation times, show that fibrils have a mean width value (i.e., 13.92 ± 3.7 nm for HeV and 17.25 ± 2.5 nm for NiV) that falls within the size range of amyloid-like fibrils (10–20 nm) [87]. Much caution should however be taken when considering these values, as they can be biased by non-constant fibril thickness and negative-staining effects.

Altogether, these data argue for the ability of the W proteins to form amyloid-like assemblies, consistent with what has been macroscopically observed in aqueous solvents. In agreement with the other experimental lines of evidence, the HeV W protein was found to have a much more pronounced fibrillation propensity, with many long fibrils being already detectable at time zero at both protein concentrations (Figure 12A) contrary to the NiV W proteins for which fibrils can only be detected at 40 µM (Figure 12). Moreover, while the fibrils formed by the HeV W protein tend to form large agglomerates, the fibrils formed by NiV W tend to form a more homogeneous layer.

## 3. Discussion

By combining in silico and experimental studies we herein show that the HeV and NiV W proteins are intrinsically disordered. The W proteins were found to be expressed as inclusion bodies in *E. coli*. This prompted us to purify them under denaturing conditions, a process often used to purify IDPs. Indeed, because IDPs lack stable secondary or tertiary structures, they remain soluble even in extreme condition of pH, temperature or salinity, with this property being even commonly exploited for their purification [88,89].

Despite their overall disordered nature, they possess some transiently populated secondary structure, particularly represented by β-strands, and some fluctuating tertiary structure. Indeed, their experimentally observed Stokes radii are closer to those expected for PMG states than to those expected for IDPs of the same size. The W proteins are more compact than expected for IDPs, a property already observed for the cognate V proteins [33]. Taking into account the high proline content of the W proteins, we first attempted at rationalizing this discrepancy by checking whether the observed hydrodynamic radii were closer to the values expected by the sequence-based model, which takes into account the proline content among other sequence features [90]. The R_S_^IDPseq^ radii calculated accordingly (see Equation (5) in Materials and Methods) were however found to be even more divergent from the experimentally observed R_S_ values, being slightly larger (58.5 Å for HeV W and 57.8 Å for NiV W) than those calculated using the simple power law model (57.4 Å for HeV W and 57.5 Å for NiV W). Therefore, the sequence determinants that dictate the relative compaction of the W proteins cannot be captured by a model that takes into account only the sequence length, the proline and the charge content. The linear distribution of proline and of charged residues, along with that of other residues whose role still remains to be uncovered, might be responsible for the higher compactness of these proteins. In particular, long-range effects, which are not captured by either the power law or the sequence-based model, may come at play especially taking into account the fact that the W_CTD_ is basic while the W_NTD_ is acidic. Moreover, and of even more relevance, intramolecular disulfide bridges appear to play a role in driving protein compaction, as judged from the fact that the addition of DTT triggers an increase in the Stokes radius.

While the R_S_-based CI of HeV W (0.40) is lower than that of HeV V (0.49), the CI of NiV W (0.43) is very close to that of NiV V (0.44). Since the R_S_-based CI of HeV NTD is equal to the CI of NiV NTD (0.51) [13,33], differences in compactness between V and W in the two viruses are to be ascribed to differences in their constituent CTDs. In the case of HeV, the higher compactness of V with respect to W can be ascribed to the fact that V_CTD_ adopts a folded Zn-finger conformation while W_CTD_ is predicted to be disordered. By contrast, it is more difficult to rationalize the similarity in the CIs of the NiV V and W proteins. If from one hand this similarity would suggest that the NiV W_CTD_ is more compact than the HeV W_CTD_, bioinformatics analyses would suggest the opposite scenario. Indeed, the HCA plot, the disorder score, as provided by CIDER (Table 1), and secondary structure predictions predict a slightly higher order content in HeV W_CTD_. On the other hand, the higher compactness of NiV W_CTD_ could arise from an enrichment in histidine residues in the NiV W_CTD_ compared to HeV W_CTD_ (Supplementary Figure S3), which could promote local and transient folding through π-π or cation-π interactions. Moreover, His residues have an enhanced sensitivity to pH, being protonated under slightly acidic conditions, which could lead to charge-driven compaction phenomena. Definite answers in this regard await direct experimental investigation of the W_CTD_ from the two proteins.

SEC experiments, as well as MS and SDS-PAGE analyses, highlighted for both proteins the existence of a disulfide-bridged dimer that may be stabilized by one of the three conserved cysteine residues. The fact that the addition of urea triggers a reduction in the elution volume of the dimer from the SEC column, argues for the presence under native conditions of residual secondary and/or tertiary structure within both the monomers building up the dimer. The chain expansion observed for the monomer under reducing conditions, supports the presence of one (or more) intramolecular disulfide bridges that play a role in driving protein compaction. Thus, in contrast with predictions provided by IUPred2A, the cysteine redox state seemingly impacts protein compaction. Specifically, the oxidized state imparts a higher compactness while the reduced state is associated with expansion of the protein chain. In addition, the oxidized state is also characterized by a higher secondary structure content, as judged from far-UV CD studies. In line with these observations, for both proteins, the R_g_ values experimentally observed under reducing conditions and in the presence of 1 M urea, exceed by ~12% the values expected for IDPs of the same length.

The critical role of the cysteines in dictating the conformational properties of the W proteins is also in line with the experimental observation that the addition of DTT in native conditions (i.e., without urea) leads to rapid, macroscopically visible, aggregation. This behavior strongly supports the involvement of disulfide bridge(s) in preventing the aggregation process of the W proteins. Whether the cysteine redox state may impact the fibrillation process as well, remains to be established and is the focus of current studies that also aim at identifying the cysteine residues involved in the formation of inter- and intramolecular disulfide bridges. Moreover, the biological relevance of a possible role of disulfide bridges in modulating W protein aggregation and fibrillation remains to be assessed in light of the effective ratio between the oxidized and reduced form in the cytoplasm and in the nucleus of infected (or transfected) cells.

CD studies in the presence of TFE unveiled a pronounced α-helical propensity in both W proteins. Please note that although TFE is known to stabilize α-helices more than β-strands, it is worth underscoring that the gain of α-helicity in the presence of TFE is not a general rule and hence truly reflects the inherent structural propensities of the protein under study. Indeed, some IDPs gain little or no α-helical content at high (i.e., 50–90%) TFE concentrations or even fold as β-sheets [91,92]. In further support of a role of TFE as a genuine secondary structure stabilizer, the intrinsically disordered N_TAIL_ domain from three paramyxoviruses undergoes α-helical folding in the presence of 20% TFE exclusively within regions known to undergo partner-induced α-helical folding [93,94]. In light of these considerations, TFE can be regarded as a *bona fide* structure stabilizer that does not promote non-native folding in IDPs. The inherent α-helical propensities of the W proteins can thus be regarded as reflecting the presence of transiently and partly structured α-MoREs, i.e., short regions prone to undergo partner-induced α-helical folding and whose transient α-helical conformation would be stabilized by TFE. In support of this, bioinformatics analyses predict many putative MoREs and α-helices within the W proteins, and their hydrodynamic radii and ellipticities values are consistent with a PMG state. Previous NMR studies focusing on NiV NTD [14,20] provide additional support pointing to the presence of transiently populated α-helices within the W proteins. On the other hand, in light of the ability of the W proteins to form amyloids, it is surprising and puzzling that TFE did not reveal any β-propensities. In this regard, it is noteworthy that although canonical amyloids have a typical cross β-sheet structure, amyloids with a cross-α architecture have been described as well [95,96]. In this latter case, amphipathic α-helices, rather than β-strands, stack perpendicular to the fibril axis and tightly self-associate into sheets to give rise to fibrillar structures with morphological and tinctorial properties similar to those of canonical cross-β amyloids. Whether amyloids formed by *Henipavirus* W proteins have a cross β-sheet or cross-α architecture remains to be established and awaits structural studies that are currently in progress in our lab.

Although CD studies unveiled a similar disorder content in the two W proteins, the NiV W protein was found to exhibit a markedly higher α-helical propensity. This difference likely arises from the higher α-helical propensity of NiV NTD with respect to HeV NTD [13,33]. Overall, the W proteins exhibit a higher α-helical propensity compared to the V proteins, where the latter were found to gain an α-helical content of ~20% at 30% TFE [33]. This difference likely arises from a propensity of the W_CTD_ to adopt an α-helical folding whereas the V_CTD_ is known to adopt a Zn-finger, β-enriched conformation [33].

**Figure 13:**
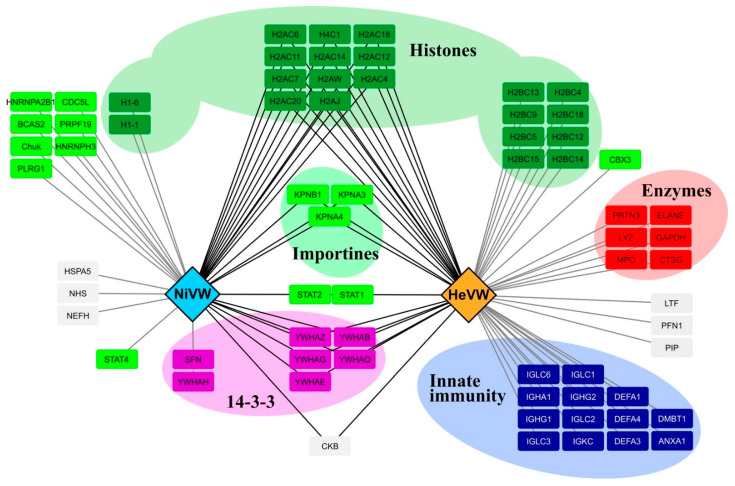
Interactome of the HeV and NiV W proteins as obtained using the INTACT database [100] and literature data mining. HeV W and NiV W are represented as blue and orange diamonds, respectively. The gene names of W protein partners are indicated in the nodes. Black lines connect HeV and NiV W with their common partners while gray lines connect them with their unique partners. Green nodes: nuclear proteins (dark green: histones, bright green: other nuclear proteins). Gray nodes: proteins without particularity.

The occurrence of partly preconfigured MoREs has been proposed to enable a more efficient start of the folding process induced by a binding partner via a reduction of the entropic cost associated with the disorder-to-order transition [49,50,61,97,98,99]. In line with this, the W proteins have a broad molecular partnership. Indeed, analysis of the interactome of the two W proteins highlighted 90 interactions in total where 53 partners have been identified for HeV W and 37 for NiV W (Figure 13 and Supplementary Table S1). All of the reported partners are human, except for the murine inhibitor of nuclear factor kappa-B kinase subunit alpha protein, whose sequence is however very close to that of its human counterpart (UniProt Accession number O15111). HeV W and NiV W share 22 common partners including several histones, importins, 14-3-3 proteins, STAT1 and STAT2 proteins and the creatine kinase B-type (Figure 13). Among the identified partners, 31 interact only with HeV W and 15 only with NiV W.

The majority of the W interacting-proteins are nuclear, mainly represented by histones. This is consistent with the presence of a nuclear localization sequence in the C-terminal part of the W proteins as well as with their reported interaction with importins [35,36]. The W proteins interact with the 14-3-3 protein family regulating apoptosis, metabolic processes and extracellular matrix organization [42], and interfering with the NF-κB-induced proinflammatory response [43]. A non-neglectable amount of protein partners (13) are implicated in innate immunity, i.e., several immunoglobulins, defensins and annexin A1. Interestingly, six enzymes possessing antimicrobial activities have been found to interact with HeV W. Finally, NiV W interacts with heterogeneous nuclear ribonucleoproteins proteins A2/B1 and H3, where A2/B1 has been found to phase separate [101].

Although the NiV W protein has a higher α-helical propensity, a higher CI (as estimated from both SEC and SEC-SAXS), and a higher predicted order content with respect to its HeV counterpart, it was unexpectedly found to exhibit a slightly higher protease sensitivity. This apparent conflicting behavior might arise from the much higher aggregation propensity of the HeV W protein that may hinder protease cleavage. This observation mirrors our previous studies where the HeV V protein was found to jellify under conditions where NiV V did not [82,83].

In line with the ability of the HeV V PNT3 region, common to both the V and W proteins, to undergo a liquid-to-gel transition and to form amyloid-like fibers [82,83], we herein show that both the HeV and NiV W proteins form phase separated condensates that bind the amyloid-specific CR and ThT dyes.

Phase separation phenomena have attracted much attention in the last decade in light of their role in driving the formation of membrane-less organellles (MLOs) (e.g., Cajal bodies, P-bodies, nucleolus, stress granules, centrosomes, aggresomes, etc.) [79]. LLPS by viral proteins has been implicated in different steps and regulatory processes in viral replication cycles (for reviews see [102,103,104]). A functionally important manifestation of LLPS driven by viral proteins is the formation of so-called “viral factories”, i.e., cytoplasmic inclusion bodies where viral replication and assembly take place. Indeed, in various *Mononegavirales* members viral factories were shown to be liquid-like MLOs resulting from LLPS of their N and P proteins [102,105,106,107,108,109,110,111,112], with IDRs playing a crucial role in this process [103]. A functionally distinct and less well-known category of LLPS is “LLPS-mediated interference with host cell functions” [103], a process that is not associated with viral replication and assembly: it rather leads to interference of host cell functions through either interaction of the viral condensates with specific host genes [113] or with cellular proteins. Interestingly, the NiV W protein was found to interact with hnRNPA2B1, a protein known to be involved in proteinopathy and amyotrophic lateral sclerosis (ALS), to exhibit an intrinsic tendency to assemble into self-seeding fibrils and to be incorporated in stress granules [114]. As such, NiV W could either interfere with the integrated stress response or recruit host stress granule components to facilitate virus replication, in line with the well-known ability of viruses to regulate stress granules [115]. Interaction of viral condensates with cellular proteins can also lead to sequestration, and hence possible inactivation, of cell proteins involved in the cell innate immune response (see [103,104] and refs therein cited). The formation of fibrillar aggregates by viral proteins, reported in a very limited number of studies, could be revisited in light of LLPS, where fibrils might correspond to solid-like inclusions formed upon maturation of liquid-like condensates. In this regard, it is worthy to note that the SARS -CoV2 nucleocapsid protein was shown to form amyloids in phase separated droplets [116].

In some out of the very few cases reporting the ability of viral proteins to form fibrillar aggregates, fibril formation was shown to be associated with various functional effects, namely (*i*) membrane disruption and cell toxicity (PB1-F2 from influenza A virus) [117], (*ii*) blockade of key cellular processes, such as necroptosis or apoptosis (RHIM-containing proteins from *Herpesviridae* members) [118,119,120], (*iii)* suppression of host cell RNA synthesis through host transcription factor sequestration in fibrillar aggregates (NSs from Rift valley fever virus) [121] and (*iv*) suppression of IFN responses (e.g., silencing of IFN-β expression and degradation of PKR) [122].

Whether the W proteins fibrillate also in the cellular context remains to be established and is the focus of ongoing efforts in our lab. Indeed, one limitation of the present study is that heterologous expression of the W proteins in *E. coli* prevents post-translational modifications (PTMs). *Henipavirus* V proteins are phosphorylated in cellula [123], and phosphorylation of IDRs is known to both trigger conformational transitions [124] and modulate phase separation and fibrillation abilities [79]. Phosphorylation is however the sole PTM that have been reported so far regarding the W proteins.

Under the assumption that fibrillation also occurs *in cellula*, it is tempting to hypothesize that the phase separation and fibrillation abilities of the *Henipavirus* V and W proteins might be related to interference with the host innate immune response. In addition, it should be kept in mind that the minimal region of the HeV V protein conferring the ability to phase separate (i.e., PNT3) [82,83] is also part of the P protein that is a constituent of the viral factories of *Mononegavirales* members [102,103,109]. Therefore, it is conceivable that the *Henipavirus* P protein may phase separate and fibrillate as well, with this property being possibly functionally coupled to the formation of viral factories.

## 4. Materials and Methods

### 4.1. Bioinformatic Analyses

Sequence alignment of the HeV W and NiV W (Malaysia strain) proteins (Uniprot code P0C1C6 and P0C1C7, respectively) was performed using Clustal Omega (https://www.ebi.ac.uk/Tools/msa/clustalo/ (accessed on 1 April 2021)). The RH-plots were generated by the PONDR server (http://www.pondr.com (accessed on 1 April 2021)). The phase diagram plots were generated using the CIDER server (http://pappulab.wustl.edu/CIDER/analysis/ (accessed on 1 April 2021)) [45]. Deviations in amino acid composition of W proteins and of their respective CTDs were computed as previously described [13] using the average amino acid frequencies of the SWISS-PROT database (as obtained from https://web.expasy.org/protscale/ pscale/A.A.Swiss-Prot.html, 2020-05 release) as the reference value. The average amino acid frequencies of the SWISS-PROT database roughly correspond to the mean composition of proteins in nature. If the average composition of an amino acid X in SWISS-PROT proteins is CSPX, and CPX is the composition of X within a protein P, deviation from the composition of X of SWISS-PROT proteins was defined for P as (CPX-CSPX)/CSPX. The HCA plots were obtained using the MeDor server [125]. Disorder predictions were obtained using the IUPred2A server [48]. MoRFs were predicted using MoRFchibi SYSTEM (https://morf.msl.ubc.ca/index.xhtml (accessed on 1 February 2021)) [57]. Secondary structure elements were predicted using the PSIPRED server (http://bioinf.cs.ucl.ac.uk/psipred/) (accessed on 1 February 2021) [58].

Structure prediction was carried out using the Phyre2 protein fold recognition server (http://www.sbg.bio.ic.ac.uk/~phyre2/html/page.cgi?id=index) (accessed on 1 April 2021) [60].

The interactome of the HeV W and NiV W proteins was built through data mining and through interrogation of the IntAct database (https://www.ebi.ac.uk/intact (accessed on 1 March 2021)) [100]) including protein partners generated by spoke expansion. IntAct is an open-source database for molecular interaction data in which all interactions are derived from literature curation or direct user submissions. The interaction network was then drawn using Cytoscape 3.8.2 [126].

### 4.2. Generation of the W Expression Constructs

The HeV and NiV (Malaysia strain) W coding sequences were built by overlapping PCR using NTD and CTD coding sequences [13]. To this end, OneTaq^®^ DNA polymerase (New England Biolabs, Ipswich, MA, USA) was used with a pDEST14 derivative containing a P gene optimized for the expression in *E. coli* as template [13]. Primers (Operon) were designed to introduce the gene fragment encoding the CTD of the W protein downstream the gene fragment encoding the NTD. Forward primer was 62 nucleotides in length and was designed to bear at its 5′ end an AttB1 site followed by a fragment encoding a Tobacco Etch virus (TEV) protease cleavage site (ENLYFQG) and by a 22-nucleotide long fragment annealing on the 5′ end of the P gene. Reverse primer was 183 nucleotide long and was designed to anneal on the last 20 nucleotides of the NTD encoding region, to bear the 132-long fragment encoding the CTD of W followed by two stop codons followed in their turn by an AttB2 site. After digestion with DpnI (New England Biolabs, Ipswich, MA, USA) to remove the methylated DNA template, the amplicons were subsequently transferred into the pDONR vector (Invitrogen, Carlsbad, CA, USA) through a Gateway BP clonase-mediated recombination reaction (Invitrogen, Carlsbad, CA, USA). From the resulting entry vectors each W gene was transferred into either the pETG-20A [127] or the pDEST17OI expression vector using Gateway LR clonase (Invitrogen, Carlsbad, CA, USA). The pETG-20A vector drives the expression under the T7 promoter of a thioredoxin (TRX) tagged fusion protein in which the protein of interest is preceded by a hexahistidine (His_6_) tag. The resulting constructs (referred to as pETG-20A/HeVW and pETG-20A/NiVW) encode fusion W proteins in which the TRX tag, the hexahistidine tag and the AttB1-encoded amino acid stretch can be cleaved off by TEV protease digestion thus leading to a protein bearing only a non-native N-terminal glycine residue. The pDEST17OI expression vector is a modified pDEST17 vector (Invitrogen, Carlsbad, CA, USA) in which LacO and LacI encoding sequences were inserted upstream the T7 promotor by swapping pDEST17 and pET-DEST42 (Invitrogen, Carlsbad, CA, USA) PshAI/XbaI fragment in order to allow a better control of protein expression [128]. The resulting constructs (referred to as pDEST17OI/HeVW and pDEST17OI/NiVW) allow the expression under the T7 promoter of N-terminally hexahistidine tagged W proteins in which the native W sequence is preceded by a vector-encoded MSYYHHHHHHLESTSLYKKAGS amino acid stretch and by a TEV cleavage site.

### 4.3. Expression and Purification of the W Proteins

The *E. coli* strain T7pRos was used for the expression of all the recombinant proteins. Cultures were grown overnight to saturation in LB medium containing 100 µg mL^−1^ ampicillin and 34 µg mL^−1^ chloramphenicol. An aliquot of the overnight culture was diluted 1/20 into 1 L of TB medium and grown at 37 °C. When the optical density at 600 nm (OD_600_) reached 0.5–0.8, isopropyl β-D-thiogalactopyranoside (IPTG) was added to a final concentration of 0.5 mM, and the cells were grown over night at either 25 °C (pETG20A-derived constructs) or 37 °C (pDEST17OI-derived constructs). The induced cells were harvested, washed and collected by centrifugation (6000× *g*, 20 min).

The W proteins fused to TRX were purified as described for the TRX-fused V proteins [33]. The pellets of bacteria transformed with pDEST17OI/W constructs were resuspended in 50 mL *per* liter of culture of buffer A1 (50 mM Tris/HCl, pH 8, 300 mM NaCl, 8 M urea) and frozen at −20 °C. Upon thawing, the cells were disrupted by sonication as described above and clarified by centrifugation. The supernatant was purified by immobilized metal affinity chromatography (IMAC) using a fast protein liquid chromatography (FPLC) Äkta system (Cytiva, Marlborough, MA, USA). The column was washed with buffer A1 supplemented with 20 mM imidazole and elution was in buffer A1 containing 250 mM imidazole. The eluents from IMAC were analyzed by SDS-PAGE and the fractions containing the protein of interest were pooled and the sample was further purified by ion exchange chromatography (IEC). To this end the sample was desalted using a HiPrep 26/10 desalting column (Cytiva, Marlborough, MA, USA) and buffer B1 (20 mM Hepes pH 7, 50 mM NaCl, 8 M urea). The fractions obtained were then loaded onto a HiPrep DEAE FF 16/10 column (Cytiva, Marlborough, MA, USA) and the protein was eluted using a linear gradient of buffer B2 (20 mM Hepes pH 7, 500 mM NaCl, 8 M urea). The fractions containing the purified recombinant proteins were stored at −20 °C in buffer B2. Prior to all ensuing analyses, urea was removed from the protein samples and the buffer exchanged using Sephadex G-25 medium columns (Cytiva, Marlborough, MA, USA). Both IMAC and IEC were performed at room temperature.

Protein concentrations were calculated using the theoretical absorption coefficients at 280 nm as obtained using the program ProtParam at the EXPASY server (http://web.expasy.org/protparam/) (accessed on 1 February 2021).

### 4.4. Mass Spectrometry

#### 4.4.1. Intact Protein Mass Analysis

Protein masses were determined on purified solution samples. To this end, the proteins in 8 M urea containing buffer were loaded onto a G-25 column (Cytiva, Marlborough, MA, USA) and eluted with 10 mM ammonium acetate. After a concentration step using Zip Tip C4 (Merck Millipore Darmstadt, Germany), 1 μL of protein at ~1.5 mg/mL was mixed with 1 μL of sinapinic acid matrix solution in 0.3% TFA/CH_3_CN (50:50 *v*/*v*). One μL of the mix was spotted on the target and analyzed by MALDI-TOF on a Ultraflex III spectrometer (Bruker Daltonics, Wissembourg, France) controlled by the Flexcontrol 3.0 package (Build 51) and operated in the linear mode, using a maximum accelerating potential of 25 kV and a 20,000–100,000 *m*/*z* range (LP_66kDa method). The laser frequency was fixed to 100 Hz and ~1000 shots per sample were cumulated. Four external standards (Protein Calibration Standard II, Bruker Daltonics) were used to calibrate each spectrum to a mass accuracy within 100 ppm. Peak picking was performed using the FlexAnalysis 3.0 software with an adapted analysis method. Parameters used were the centroid peak detection algorithm, S/N threshold fixed to 5 and a quality factor threshold of 30.

#### 4.4.2. Peptide Mass Fingerprintings

Protein bands were excised from SDS-PAGE and digested overnight at 37 °C with high-sequencing-grade trypsin (Promega, Madison, WI, USA) after cysteine reduction in the presence of 10 mM DTT and alkylation with 50 mM iodoacetamide. Peptide extracts were pooled and dried in a centrifugal vacuum system. Samples were reconstituted with 0.1% trifluoroacetic acid in 4% acetonitrile and analyzed by LC-MS/MS using an Orbitrap QExactive Plus Mass Spectrometer (Thermo Electron, Bremen, Germany) online with an Ultimate 3000RSLCnano chromatography system (Thermo Fisher Scientific, Sunnyvale, CA, USA). Peptides were separated on a Dionex Acclaim PepMap RSLC C18 column. First, peptides were concentrated and purified on a pre-column in solvent A (0.1% formic acid (FA) in 2% acetonitrile). In the second step, peptides were separated on a reverse-phase LC EASY-Spray C18 column from Dionex (PepMap RSLC C18, 50 cm × 75 µm I.D, 100 Å of pore size, 2 µm of particle size) at 300 nL/min flow rate. After column equilibration using 4% of solvent B (20% water–80% acetonitrile–0.1% FA), peptides were eluted by a two step linear gradient (4–20% acetonitrile/H2O; 0.1% FA for 90 min and 20–45% acetonitrile/H2O; 0.1% FA for 30 min). For peptide ionization in the EASY-Spray nanosource, spray voltage was set at 2.2 kV and the capillary temperature at 275 °C. The Orbitrap QExactive was used in data-dependent mode to switch consistently between MS and MS/MS. MS spectra were acquired with the Orbitrap in the range of *m*/*z* 400–1600 at a FWHM resolution of 120,000 measured at 400 *m*/*z*. AGC target was set at 4.0 × 10^5^with a 50 ms of maximum injection time. The more abundant precursor ions were selected and CID fragmentation was performed in the ion trap to have maximum sensitivity. The number of precursor ions was automatically defined with a maximum injection time of 300 ms. The signal threshold for an MS/MS event was set to 5000 counts. Charge state screening was enabled to exclude precursors with 0 and 1 charge states. Dynamic exclusion was enabled with a repeat count of 1 and a duration of 60 s. The acquired raw data were processed using Proteome Discoverer (version 1.4.1.14, Thermo Fisher Scientific). Spectra were searched using SEQUEST (Thermo Fisher Scientific) against a homemade database comprising 20,150 human and 4306 *E. coli* sequences and the HeV W and NiV W sequences both with and without the initial methionine. Search parameters were: (i)trypsin; two miscleavages allowed; (ii) mass tolerance of 6 ppm for monoisotopic precursor ions and 0.8 ppm for fragment ions from MS/MS; (iii) Cys carbamidomethylation (+57.02146 Da) as a fixed modification; Met oxidation (+15.99491 Da) and N-terminal acetylation (+42.0106 Da) as variable modifications; (iv) minimum peptide length of four residues. Only high-score peptides were selected. Proteins were identified with a false discovery rate (FDR) of 1%.

### 4.5. Estimation of the Hydrodynamic Radius by SEC

The hydrodynamic radii (Stokes radii, R_S_) of the proteins were estimated by analytical SEC using a Superose 6 Increase 10/300 column (Cytiva, Marlborough, MA, USA). Hepes Buffered Saline (HBS, 20 mM Hepes pH 7, 150 mM NaCl) was used as elution buffer unless differently indicated. Typically, 250 μL of purified protein at 2.5 mg mL^−1^ were injected.

The Stokes radii of proteins eluted from the SEC column were deduced from a calibration curve obtained using globular proteins of known R_S_ (Thyroglobulin: 85 Å, Ferritin: 61 Å, Aldolase: 48.1 Å, Conalbumin: 36.4 Å, Carbonic anhydrase: 23 Å, RNAse A: 16.4 Å and Aprotinin: 13.5 Å)

The R_S_ (in Å) of a natively folded (Rs^NF^), fully unfolded state in urea (R_S_^U^) and natively unfolded premolten globule (PMG) (R_S_^PMG^) protein with a molecular mass (MM) (in Daltons) were calculated according to [47]:

log (R_S_^NF^) = 0.357 × (log MM) − 0.204 (1)

log (R_S_^U^) = 0.521 × (log MM) − 0.649 (2)

log (R_S_^PMG^) = 0.403 × (log MM) − 0.239 (3)

The Rs^NF^, R_S_^U^ and R_S_^PMG^ expected for a natively folded, and fully unfolded and PMG dimeric form of a protein with molecular mass MM were calculated according to Equations (1)–(3) by replacing (log MM) with (log 2 × MM).

The R_S_ of an IDP with N residues was also calculated according to [90] using either the simple power law model:

R_S_^IDP^ = R_0_N^ν^ (4)

where R_0_ = 2.49 and ν = 0.509, or the sequence-based model:

R_S_^IDPseq^ = (AP_Pro_ + B) × (C|Q| + D) × S_his_ × R_0_N^ν^ (5)

where A = 1.24, P_Pro_ = fractional proline content, B = 0.904, C = 0.00759, |Q| = absolute value of the difference between the total number of negatively charged and positively charged residues, D = 0.963, S_his_ = 0.901 (correction factor for histidine tag).

The compaction index (CI) is expressed as according to [129]:

CI = (R_s_^U^ − R_s_^obs^)/(R_s_^U^ − R_s_^NF^) (6)

This parameter, which allows comparison between proteins of different lengths, in principle varies between 0 and 1, with 0 indicating minimal compaction and 1 maximal compaction.

### 4.6. Protease Sensitivity Assay

Protein samples in 50 mM Tris/HCl, pH 8, 150 mM NaCl buffer at a final concentration of 50 μM were incubated at room temperature with 2.5 μg mL^−1^ thermolysin (Sigma Aldrich, Saint Louis, MO, USA) in a total reaction volume of 55 μL. The extent of proteolysis was analyzed by 15% SDS-PAGE on 5-μg samples withdrawn from the reaction mixture at specified time intervals after the reaction start. The reaction was stopped by mixing each sample with Laemmli electrophoresis buffer and immediately boiling for 5 min. Bovine serum albumin (BSA) (New England Biolabs, Ipswich, MA, USA) was digested in the same manner and used as a control.

### 4.7. Differential Scanning Fluorimetry (DSF)

The DSF analysis of the W proteins was carried out in the presence of a fluorescent dye as described in [130,131]. The experiment was conducted using a PCR instrument (Biorad) and 96-well plates containing 25 μL of mixture per well. Each well contained 21.5 μL of HeV W or NiV W at 1 mg mL^−1^ in HBS and 3.5 μL of SYPRO Orange (ThermoFisher Scientific 7× solution in water (prepared from a 5000× stock solution in DMSO). Fluorescent signals were acquired with excitation and emission wavelengths at 485 nm and 625 nm, respectively. Temperature scans were performed from 20 °C to 95 °C.

### 4.8. Circular Dichroism

Far-UV CD spectra were measured using a Jasco J-810 dichrograph (Hachiōji, Japan), flushed with N_2_ and equipped with a Peltier thermoregulation system set at 20 °C. One-mm thick quartz cuvettes were used. Proteins concentrations were 1 μM. Spectra were measured between 260 and 190 nm with a scanning speed of 50 nm/min and a data pitch of 0.2 nm. Response time was set to 4 s and the bandwidth to 2 nm. CD spectra were recorded in 10 mM sodium phosphate pH 7 either in the absence or in the presence of 1 M urea supplemented or not with 10 mM DTT. Each spectrum is the average of ten acquisitions. The spectrum of buffer was subtracted from the protein spectrum. Spectra were smoothed using PRISM.

Far-UV CD spectra were also recorded in the presence of increasing concentrations of TFE (from 10 to 50% *v*/*v*). The midpoints of transition and *m* values were derived upon fitting the data to a sigmoid curve using PRISM.

Mean molar ellipticity values per residue (MRE) were calculated as

[θ] = 3300*m*∆A/*lcn* (7)

where *l* is the path length in cm, *n* is the number of residues, *m* is the molecular mass in Daltons and *c* is the concentration of the protein in mg mL^−1^. Numbers of amino acid residues are 476 for HeV W and 478 for NiV W. Molecular masses are 52,706 Da for HeV W and 52,875 Da for NiV W.

The DICHROWEB website (http://dichroweb.cryst.bbk.ac.uk/html/home.shtml) (accessed on 1 April 2021), which was supported by grants to the BBSRC Centre for Protein and Membrane Structure and Dynamics (CPMSD) [132], was used to analyze the experimental data in the 190–260 nm range using unsmoothed, subtracted spectra. The content in the various types of secondary structure was estimated using the CDSSTR deconvolution algorithm with the reference protein set 7.

### 4.9. Small-Angle X-ray Scattering (SAXS)

Samples at 5 mg mL^−1^ in HBS containing 8 M urea were loaded onto a G-25 column (Cytiva, Marlborough, MA, USA) and eluted with freshly prepared HBS supplemented with 1 M urea and 5 mM DTT. The final protein concentration was 2.35 mg mL^−1^ for HeV W and 2.47 mg mL^−1^ for NiV W. Synchrotron X-ray scattering data were collected at SOLEIL (Gif-sur-Yvette, France) on the SWING beamline at a working energy of 12 KeV. Data were collected using a Dectris EIGER 4M detector at a sample-to-detector distance of 2.0 m and a wavelength (λ) of 1.03324 Å. This setup leads to scattering vectors (*q*) between 4.56 × 10^−4^ and 0.540 Å^−1^ (with *q* = 4π sin(θ)/λ, where 2θ is the scattering angle).

The calibration was made with water. For both proteins 70 μL were injected onto a BioSec 3–300 SEC column (Agilent, Santa Clara, CA, USA). The elution buffer was HBS, 1 M urea, 5 mM DTT. The flow rate was 0.2 mL min^−1^ and the temperature was 20 °C. The exposure was in the continuous mode, with 1 frame sec^−1^ (990 ms exposure and 10 ms dead time). Data reduction and frames subtraction were done manually with the beamline software FOXTROT. Buffer blank frames were taken in the dead volume of the column.

Buffer-subtracted frames were submitted to Gaussian decompositions using the UltraScan solution modeler (US-SOMO) HPLC-SAXS module [133]. Deconvoluted data corresponding to the W proteins were compared with the CorMap test and averaged. The final scattering intensities were analyzed using the ATSAS program package [134]. Linearity in the Guinier region was used to exclude sample aggregation. The few data points differing from the Guinier fitting at low angles were deleted and the useful data range was determined with SHANUM [134]. The radius of gyration (R_g_) was estimated at low angles (*q* < 1.3/R_g_) according to the Guinier approximation [135,136]:

(8) $$\mathrm{lnI}\left(q\right)={\mathrm{lnI}}_{0}-\frac{\left(q^{2}{\;R}_{g}^{2}\right)}{3}$$

The pair-distance distribution function, P(r), from which the D_max_ and the R_g_ were estimated, was computed using GNOM and manually adjusted until a good CorMap *p*-value (α > 0.01) was obtained [137].

The theoretical R_g_ value (in Å) expected for various conformational states was calculated using Flory’s equation

R_g_ = R_0_N^ν^ (9)

where N is the number of amino acid residues, R_0_ a constant and ν a scaling factor. For IDPs, R_0_ is 2.54 ± 0.01 and ν is 0.522 ± 0.01 [138], for chemically denatured (U) proteins R_0_ is 1.927 ± 0.27 and ν is 0.598 ± 0.028 [138], and for natively folded (NF) proteins R_0_ = √(3/5) × 4.75 and ν = 0.29 [139].

As in the case of the R_s_, the CI allows comparing the degree of compaction of a given IDP, through comparison of the observed R_g_ to the reference values expected for a fully unfolded and a folded conformation of identical mass. The CI referred to the R_g_ can be calculated as follows [129]:

CI = (R_g_^U^ − R_g_^obs^)/(R_g_^U^ − R_g_^NF^) (10)

where R_g_^obs^ is the experimental value for a given protein, and R_g_^U^ and R_g_^NF^ are the reference values calculated for a fully unfolded (U) and natively folded (NF) form as described above. Akin the R_S_-based CI, this index increases with increasing compaction.

The flexibility of the proteins was assessed with the dimensionless Kratky plot ((*q*R_g_)^2^ I(*q*)/I_0_ *vs q*R_g_) and the Krakty-Debye plot (*q*^2^I(*q*) *vs q*^2^).

Modeling of the W proteins as conformational ensembles was done with the program suite Ensemble Optimization Method (EOM) 2.0 [140] using the default parameters and random coil conformers. EOM first generates an initial pool of 10,000 random, Cα-only conformers from the amino acid sequence. Subsequently, from this pool EOM 2.0 generates a conformational sub-ensemble that best fits the experimental SAXS data. To minimize over-fitting, EOM attempts at minimizing the number of conformers able to fit the experimental data and usually generates ensembles consisting of 5 to 40 conformers. For both W proteins, the amino acid sequence provided as imput to EOM was that of the recombinant W protein without the initial methionine, which was found to be cleaved off. Gaussian distributions of R_g_ and D_max_ of the generated conformers were automatically calculated as well as their respective theoretical scattering profile. EOM uses a genetic algorithm (GA) to select an ensemble that best fits the experimental scattering curve. From the width of the R_g_ distribution the flexibility of the particles can be extracted, whereby a narrow distribution indicates a rather rigid particle and broader distributions indicate higher flexibility. Using EOM 2.0, systematic quantification of the flexibility was made using the metric Rflex—which computes the Shannon information entropy of the distributions [140]. Experimental error-independent goodness-of-fit was also confirmed using the software CorMap that estimates the differences between one-dimensional spectra independently of explicit error estimates, using only data point correlations [72].

SEC-SAXS data have been deposited in the Small-Angle Scattering Biological Data Bank (SASBDB) [141] under codes SASDLK9 and SASDLL9 for the set of data of HeV W and of NiV W, respectively. The HeV and NiV W ensembles derived using SEC-SAXS data have been deposited within the Protein Ensemble Database (PED-DB, https://proteinensemble.org/) (accessed on 14 December 2021) [142] under accession numbers PED00204 and PED00205, respectively.

### 4.10. SDD-AGE Analysis, and Congo Red and Thioflavin T Binding Assays

Semi-Denaturing Detergent-Agarose Gel Electrophoresis (SDD-AGE) analysis was performed on a 1.8% agarose (7 cm × 14 cm) gel in 0.1% SDS, Tris, Acetate, EDTA (TAE) buffer. Both HeV and NiV W (70 μM in HBS, pH 7) were incubated at either 4 °C or 37 °C for 48 h. Samples were then incubated for 10 min at 37 °C with SDD-AGE loading buffer (TAE 2×, 20% glycerol, 8% SDS, 1% Bromophenol blue) and loaded onto gels (60 μL each). A sample of conalbumin (75 kDa, (Cytiva, Marlborough, MA, USA) pre-heated at 65 °C for 30 min, was processed in the same way and used as a control. The electrophoretic run was performed at low voltage (3 V/cm gel length) in ice using 0.1% SDS, TAE as running buffer. The gel was stained with EZBlue™ Gel Staining Reagent (Sigma Aldrich, Saint Louis, MO, USA).

Congo Red (CR, Sigma Aldrich, Saint Louis, MO, USA) was used for both qualitative and quantitative binding assays. In both case a stock solution of CR at 50 μM in Phosphate Buffer Saline (PBS, 137 mM NaCl, 2.7 mM KCl, 10 mM Na_2_HPO_4_, 1.8 mM KH_2_PO_4_, pH 7.4), 10% ethanol, 0.01% sodium azide was prepared. Qualitative binding assays were performed using the dye at a final concentration of 18 μM in the presence of either HeV W or NiV W at 75 μM in 50 mM sodium phosphate buffer at pH 7.2 in a final volume of 50 μL. The protein samples were previously incubated 3 days at room temperature to enable aggregates to form. At the end of the incubation, CR was added and incubated for 10 min. The samples were then centrifuged, and the sedimented condensate was washed three times with 50 μL of sodium phosphate buffer.

Quantitative, spectrophotometric measurement of CR binding was carried out using the dye at a final concentration of 5 μM in the presence of either HeV W or NiV W at 20 μM in 50 mM sodium phosphate buffer at pH 7.2 in a final volume of 100 μL. The adsorption spectrum was recorded after 27 h of incubation at either RT or 37 °C using a NanoDrop ND-1000 (Thermo Scientific, Waltham, MA, USA) spectrophotometer in the 400–700 nm range. Five μM CR in sodium phosphate buffer without the W protein was used as a control. Following incubation for 27 h at 37 °C, the W samples were also heated at 95 °C for five minutes. In all experiments, the dye was added after the incubation and prior to spectrophotometric measurements. The measles virus N-Tail protein, i.e., an IDP with no propensities to fibrillate [143], was used as a negative control.

The fluorescent dye Thioflavin T (ThT, Sigma Aldrich, Saint Louis, MO, USA) was used to monitor the aggregation kinetics of NiV and HeV W. A stock solution at 2 mM in HBS pH 7 was used. The proteins (40 μM in HBS pH 7) were incubated in the presence of 50 μM ThT for five minutes (equilibration time) before following the aggregation process by fluorimetry for up to two hours. Following incubation for two hours, the samples were also heated at 95 °C for five minutes and fluorescence emission measured again. Fluorescence measurements were carried out on a Cary Eclipse Fluorescence Spectrophotometer (Agilent, Santa Clara, CA, USA) equipped with a Peltier temperature controller set on 37 °C. ThT was excited at 440 nm (slitwidth: 5 nm) and fluorescence emission was recorded between 470 and 520 nm (slitwidth: 10 nm) every two minutes. Spectra were recorded with a scan rate of 300 nm/min with a data interval of 0.5 nm and an averaging time of 0.1 s. The measles virus N-Tail protein was used as a negative control in the same conditions. As an additional control, the fluorescence of a sample containing solely ThT was monitored and found not to increase over time.

### 4.11. Negative-Staining Transmission Electron Microscopy (TEM)

Two samples at different concentrations (10 and 40 µM) of NiV and HeV W were prepared and analyzed at different times to monitor their evolution. Incubation was carried out at 37 °C in HBS pH 7. Prior to each measurement, the samples were diluted to reach a final concentration of 1.6 µM. Drops of 3 μL of the diluted solution were deposited onto a glow discharge carbon coated grid (Carbon 300 mesh 3 mm Cu, TAAB). Prior to protein deposition, grids were exposed to plasma glow discharge for 20 s using a GloQube (Quorum, Lewes, UK) (Current 25 mA) in order to increase protein adhesion. The grids were washed three times with 50 µL of buffer, then washed in 35 µL 2% (*w*/*v*) Uranyl Acetate solution (LauryLab, Brindas, France) before incubating them for 1 min in the latter solution. Excess of uranyl was blotted and grids were dried for 1 h at RT. Images were collected using a TECNAI T12 Spirit microscope (Thermo Fisher Scientific, Waltham, MA, USA) operated at 120 kV, and a Veleta 2 K × 2 K CCD camera (Olympus, Shinjuku, Tokyo, Japan).

## 5. Conclusions

This study reports the characterization of the conformational properties of the *Henipavirus* W proteins, two key proteins in the host-pathogen race for which molecular data were hitherto conspicuously lacking. Compelling experimental evidence of their disordered nature and ability to fibrillate was obtained. The disordered nature of the W proteins likely endows them with several functional advantages such as (i) ability to interact with a large panel of (host) proteins, (ii) increased exposure of PTM sites, (iii) modulation of interaction strength and accessibility of binding sites through PTMs, (iv) alleviation of structural constraints on overlapping coding regions and mutational robustness (see also [14]), and (v) ability to phase-separate and to form amyloid-like fibrils in the condensed phase.

Notably, W fibrils are formed in near physiological conditions (salt, pH) and at a low protein concentration, thus advocating for a physiologically relevant phenomenon. The present study provides an additional example, among the few reported so far, of a viral protein forming amyloid-like fibrils, therefore significantly contributing to enlarge our currently limited knowledge of viral amyloids.

The present results constitute an asset for further investigating the ability of the *Henipavirus* W proteins to also form fibrils in the cellular context and for unraveling their functional impact. In light of the molecular partnership of the W proteins, our working hypothesis is that the W fibrillar condensates might interfere with the host innate immune and inflammatory response. In the long term, these studies, which are currently ongoing in our lab, are expected to contribute to shed light onto the molecular mechanisms of *Henipavirus* pathogenesis and pave the way to the design of new antiviral strategies aimed at abrogating the ability of these viruses to escape the innate immune response.

## Figures and Tables

**Table 1:** Properties of the amino acid sequences of the W proteins from HeV and NiV.

Proteins	N	pI	f_+_	f_-_	FCR	NCPR	k	Hydropathy	Disorder Promoting	PDR
HeV W	448	4.93	0.123	0.170	0.292	−0.047	0.223	3.545	0.721	2
HeV W_NTD_	404	4.64	0.111	0.186	0.297	−0.074	0.200	3.500	0.730	2
HeV W_CTD_	44	11.89	0.227	0.023	0.250	0.205	0.209	3.952	0.636	2
NiV W	450	4.84	0.117	0.169	0.287	−0.051	0.194	3.674	0.696	2
NiV W_NTD_	406	4.53	0.106	0.183	0.288	−0.076	0.167	3.677	0.697	2
NiV W_CTD_	44	11.89	0.227	0.046	0.273	0.182	0.292	3.645	0.682	2

N: residue number; pI: isoelectric point; f_+_: fraction of positively charged residues; f_-_: fraction of negatively charged residues; FCR: fraction of charged residues (f_+_ + f_−_); NCPR: net charge per residue, value of the difference between the fraction of positively charged and negatively positively residues, (|f_+_ − f_−_|); k: linear distribution of opposite charges; Hydropathy: average Kyte-Doolittle hydropathy value rescaled to lie between 0 (least hydrophobic) and 9 (most hydrophobic); Disorder promoting: fraction of residues falling in the disorder promoting category; PDR: phase diagram region. All parameters were obtained using CIDER (http://pappulab.wustl.edu/CIDER/ (accessed in 1 April 2021) [45].

**Table 2:** Stokes radii (R_S_^obs^, Å) of the major SEC peak and expected values for the various conformational states.

Proteins	Mass	R_S_^obs^	R_S_^NF^	R_S_^PMG^	R_S_^U^	R_S_^IDP^	R_S_^obs^/R_S_^NF^	R_S_^obs^/R_S_^PMG^	R_S_^obs^/R_S_^U^	R_S_^obs^/R_S_^IDP^	CI
HeV W	52,706	51.0 ± 0.9	30.3	46.1	64.7	57.4	1.68	1.11	0.79	0.89	0.40 ± 0.03
NiV W	52,875	50.1 ± 0.8	30.3	46.2	64.8	57.5	1.65	1.08	0.77	0.87	0.43 ± 0.03

R_S_^obs^: experimentally observed Stokes radius (mean value and s.d. from three independent experiments); R_S_^NF^: R_S_ expected for a natively folded (NF) form; R_S_^PMG^: R_S_ expected for a premolten globule (PMG); R_S_^U^: R_S_ expected for a fully unfolded form; R_S_^IDP^: R_S_ expected for an IDP based on the simple power law model; Mass: molecular mass (Daltons) calculated from the amino acid sequence of the recombinant protein. CI: compaction index.

**Table 3:** SAXS-derived parameters.

Proteins	I(0) cm^−1^	R_g_ (Å) Guinier	R_g_ P(r) (Å)	R_g_ (ave) EOM (Å)	D_max_ (Å)	Rflex (%) (pool)	Rflex (%) (ens)	χ2	*p*-Value CorMap	R_g_^IDP^ (Å)	CI
HeV W	0.030 ± 2.4 × 10^−4^	72.01 ± 0.89	73.62 ± 0.40	71.7	240	84.7	82.5	0.52	0.530	63.5	0.089 ± 0.016
NiV W	0.024 ± 3.1 × 10^−4^	70.96 ± 1.27	73.62 ± 0.62	70.7	245	84.5	82.0	0.44	0.014	63.6	0.111 ± 0.023

I(0): Intensity at zero angle as determined from Guinier approximation; R_g_ Guinier: R_g_ values as obtained from Guinier approximation; R_g_ P(r): R_g_ as obtained from pairwise distance distribution; R_g_ (ave) EOM: average value in the final EOM ensemble; D_max_: maximal intramolecular distance from P(r); Rflex (pool): flexibility index in the initial pool; Rflex (ens): flexibility index in the final ensemble; χ2: quality of the fit between experimental and back-calculated data from the EOM ensemble; *p*-value: quality of the fit between experimental and back-calculated data from the EOM ensemble, as provided by CorMap. R_g_^IDP^: R_g_ expected for an IDP based on the simple power law model; CI: compaction index.

## Data Availability

The data present in the current study are available from the corresponding author on reasonable request.

## Supplementary Materials

The following are available online at https://www.mdpi.com/article/10.3390/ijms23020923/s1.

## Appendix A

The hydrophobic cluster analysis (HCA) provides a graphical representation of the sequence that enables identifying disordered regions [56]. Although HCA was not originally intended to predict disorder, it is very useful for unveiling disordered regions. HCA outputs can be obtained from https://mobyle.rpbs.univ-paris-diderot.fr/cgi-bin/portal.py#forms::HCA. HCA (accessed on 1 April 2021) provides a two-dimensional helical representation of protein sequences in which hydrophobic clusters are plotted along the sequence. Glycine residues are represented as diamonds, prolines as stars, threonines as squares and serines as squares containing a dot. Acidic and basic residues are shown in red and in blue, respectively. Hydrophobic residues are shown in green. Disordered regions are recognizable as they are depleted in (or devoid of) hydrophobic clusters. HCA also enables identifying short regions with propensity to fold that appear as regions locally enriched in small hydrophobic clusters within regions otherwise devoid of such clusters.
