# Supplementary material for: Experimental Evidence of Intrinsic Disorder and Amyloid Formation by the Henipavirus W Proteins
Source: Int J Mol Sci. 2022 Jan 15;23(2):923. doi: 10.3390/ijms23020923 (PMC8780864; doi:10.3390/ijms23020923)
Supplement: Supplementary file 1 [file ijms-23-00923-s001.zip › ijms-1534241-supplementary.pdf]

International Journal of *Molecular Sciences*

MDPI

Supplementary Information

Experimental evidence of intrinsic disorder and amyloid formation by the Henipavirus W proteins

Giulia Pesce[1¶], Frank Gondelaud[1¶], Denis Ptchelkine[1], Juliet F. Nilsson[1], Christophe Bignon[1], Jérémy Cartalas[1], Patrick Fourquet[2] and Sonia Longhi[1*]

[1]Lab. Architecture et Fonction des Macromolécules Biologiques (AFMB), UMR 7257, Aix Marseille University and Centre National de la Recherche Scientifique (CNRS), 163 Avenue de Luminy, Case 932, 13288 Mar-seille CEDEX 09, France.

[2]INSERM, Centre de Recherche en Cancérologie de Marseille (CRCM), Centre National de la Recherche Scientifique (CNRS), Marseille Protéomique, Institut Paoli-Calmettes, Aix-Marseille University, 27 Bvd Leï Roure, CS 30059, 13273 Marseille CEDEX 09, France.

[¶]These authors have equally contributed to the work.

*to whom correspondence should be sent

Sonia Longhi

AFMB, UMR 7257 CNRS and Aix-Marseille University

163, avenue de Luminy, Case 932, 13288 Marseille Cedex 09, France

Tel: (33) 4 91 82 55 80; Fax: (33) 4 91 26 67 20

E-mail: sonia.longhi@univ-amu.fr

Supplementary Figures S1 to S7

Supplementary Table S1

```
HeVW   MDKLDLVNDGLDIIDFIQKNQKEIQKTYGRSSIQQPSTKDRTRAWEDFLQSTSGEHEQAEGGMPKNDGGTEGRNVEDLSSVTSSDGTIGQ     90
NiVW   MDKLELVNDGLNIIDFIQKNQKEIQKTYGRSSIQQPSIKDQTKAWEDFLQCTSGESEQVEGGMSKDDGDVERRNLEDLSSTSPTDGTIGK     90
       ****:******:************************ **:*:*******.**** **.* *** *:**..* **:*****.: :*****:

HeVW   RVSNTRAWAEDPDDIQLDPMVTDVVYHDHGGECTGHGPSSSPERGWSYHMSGTHDGNVRAVPDTKVLPNAPKTTVPEEVREIDLIGLEDK    180
NiVW   RVSNTRDWAEGSDDIQLDPVVTDVVYHDHGGECTGYGFTSSPERGWSDYTSGANNGNVCLVSDAKMLSYAPEIAVSKEDRETDLVHLENK    180
       ****** ***. *******:***************:* :******** : **:::***  * *:*:*  **: :* :* ** **: **:*

HeVW   FASAGLNPAAVPFVPKNQSTPTEEPPVIPEYYYGSGRRGDLSKSPPRGNVNLDSIKIYTSDDEDENQLEYEDEFAKSSSEVVIDTTPEDN    270
NiVW   LSTTGLNPTAVPFTLRNLSDPAKDSPVIAEHYYGLGVKEQNVGPQTSRNVNLDSIKLYTSDDEEADQLEFEDEFAGSSSEVIVGISPEDE    270
       ::::****:****. :* * *::: *** *:*** * : :        ********:******: :***:***** *****::. :***:

HeVW   DSINQEEVVGDPSDQGLEHPFPLGKFPE----KEETPDVRRKDSLMQDSCKRGGVPKRLPMLSEEFECSGSDDPIIQELEREGSHPGGSL    356
NiVW   EPSSVGGKPNESIGRTIEGQSIRDNLQAKDNKSTDVPGAPKD----SAVKEEPPQKRLPMLAEEFECSGSEDPIIRELLKENSLINCQQ    356
       :  .     .:  .: :*      .::      . :.*..  **    .: *.    ******:********:****:** :*.*  . .

HeVW   RLR-EPPQS-SGNSRNQPDRQLKTGDAASPGGVQRPGTPMPKSRIMPIKKGAQTRSLNMLGRKTCLGRRVVQPGMFADYPPTKKARVLLRRMSN  448
NiVW   GKDAQPPYHWSIERSISPDKTEIVNGAVQTADRQRPGTPMPKSRGIPIKKGAQTRNIHLLGRKTCLGRRVVQPGMFEDHPPTKKARVSMRRMSN  450
           :**   * :   .**:   ...*.. .. *********** :*********.:::***************** *:********* :*****
```

Figure S1. Amino acid sequence alignment of HeV and NiV W proteins. Basic residues are in blue, acidic residues in red, prolines in pink, cysteines in green and aromatic residues in orange. Conserved cysteines are shown in bold and underlined. The alignment was generated with Clustal Omega. Asterisks denote identical residues, and colons similar residues.

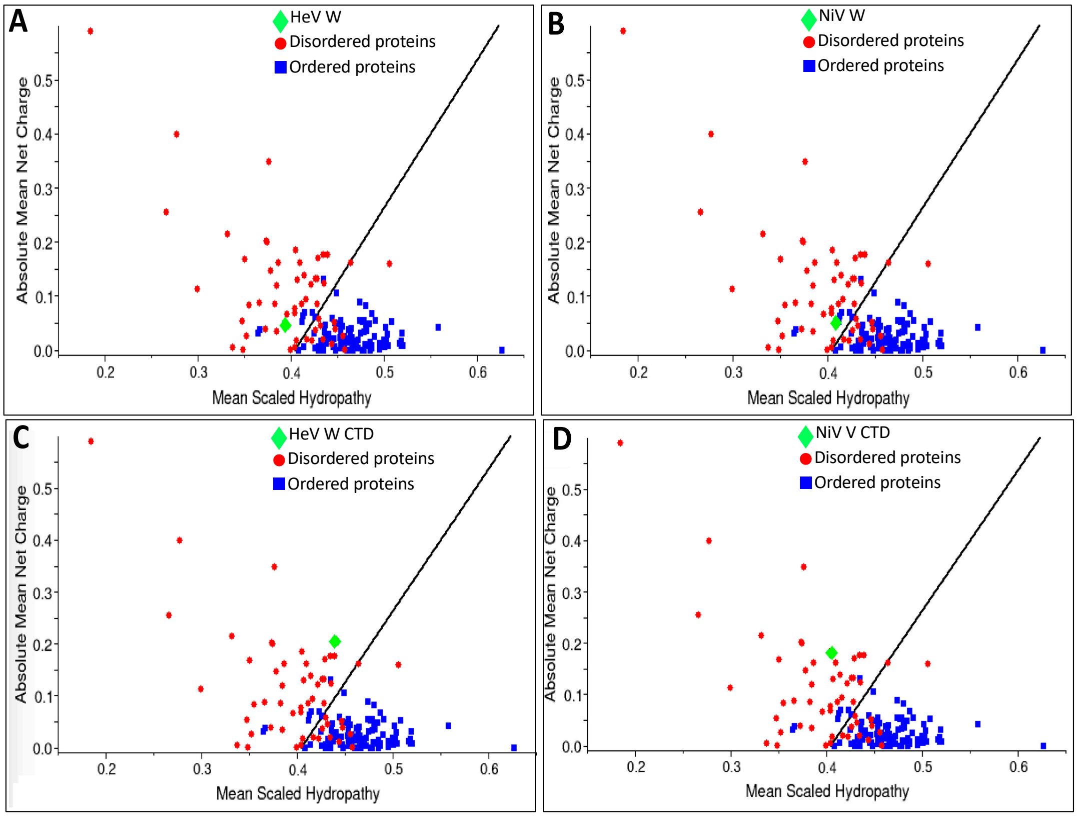

Figure S2. Charge-hydropathy plot of the HeV (**A**) and NiV (**B**) W proteins and of their CTDs (**C**,**D**). The mean net charge (R) is plotted against the mean hydrophobicity (H). In the left part of the CH plot, a protein is predicted to be intrinsically disordered, whereas it is predicted to be structured if it falls in the right part of it (see Materials and Methods).

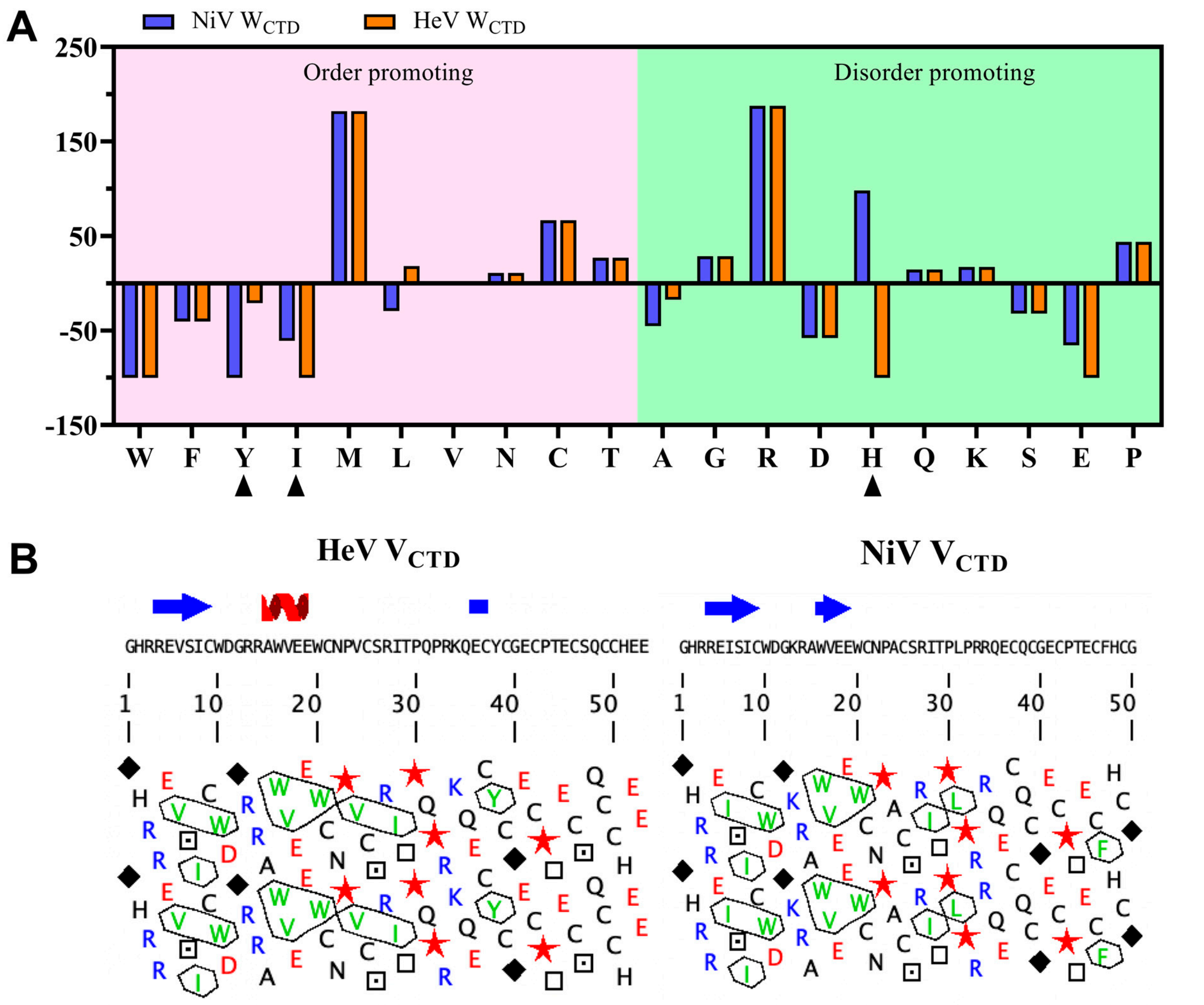

Figure S3. (A) Deviation in amino acid composition from the Swiss-PROT database of the HeV and NiV C-terminal domains (CTDs). The relative enrichment in disorder promoting and depletion in order-promoting residues is shown. Residues have been ordered on the x-axis according to the TOP-IDP flexibility index as described in [1]. Arrowheads point residues whose abundance differs most between the two viruses. (**B**) HCA plots of the CTD of the HeV and NiV V proteins featuring the amino acid sequence above the plot along with secondary structure elements as predicted by the Pred2ary program implemented in MeDor [2].

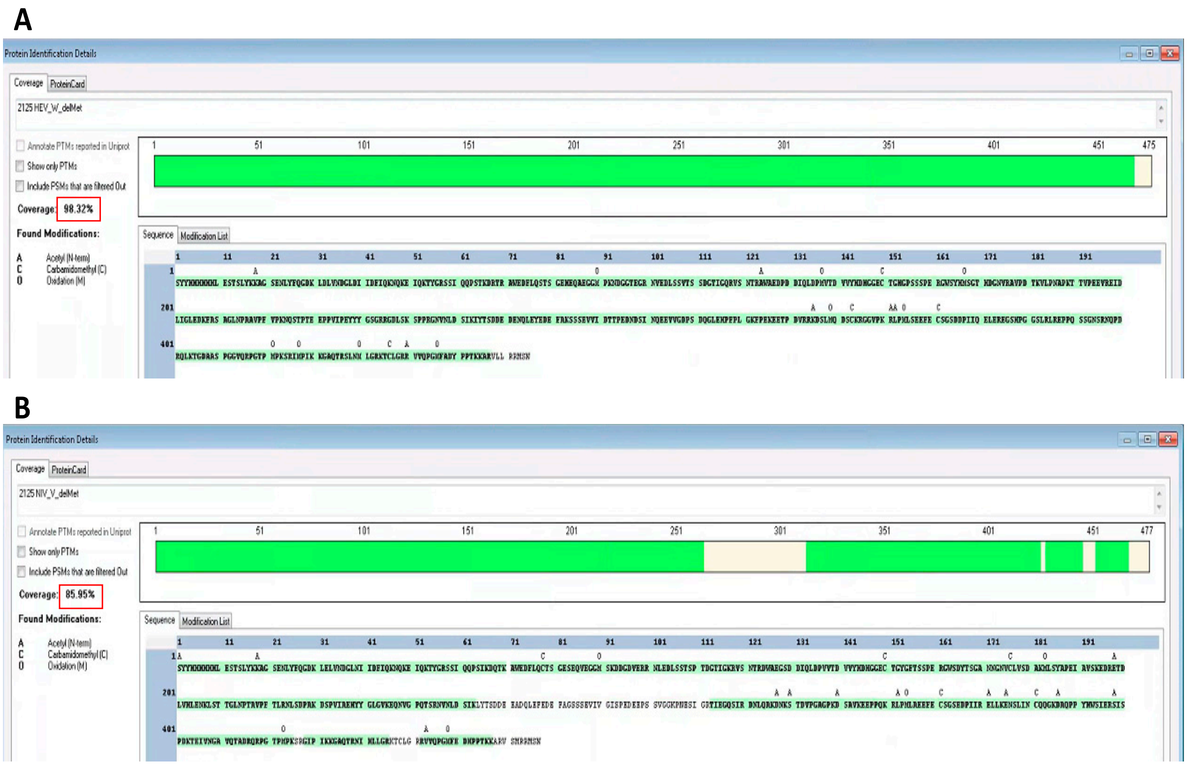

Figure S4. Results of Peptide Mass Fingerprint (PMF) of HeV (**A**) and NiV (**B**) W proteins. Peptides obtained by tryptic enzymatic digestion are shown in green. The sequence coverage is ~98% for HeV W and ~86% for NiV W (red frame).

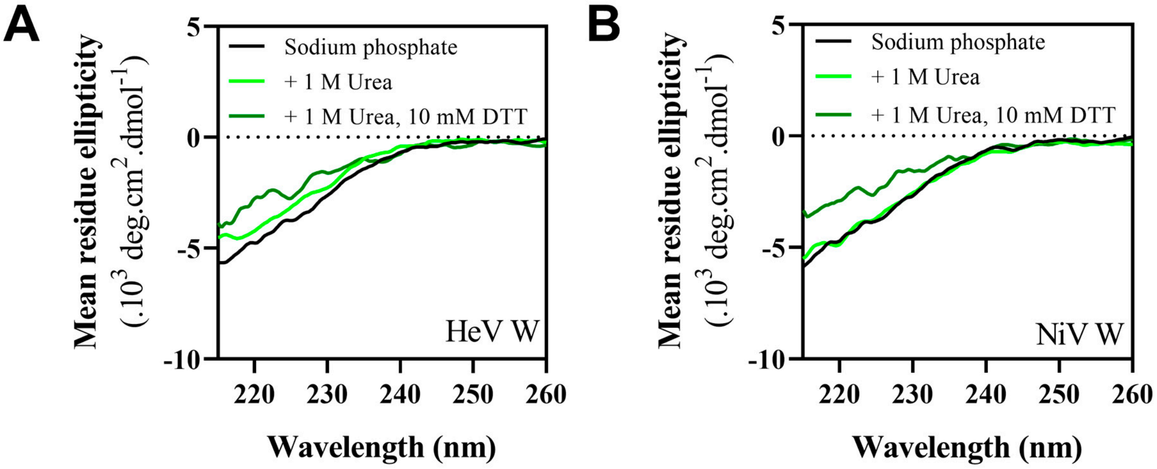

Figure S5. Far-UV CD spectra of HeV (**A**) and NiV (**B**) W either in 10 mM sodium phosphate at pH 7 or in buffer supplemented with 1 M urea or 1 M urea, 10 mM DTT. The proteins were at 1 µM. Spectra were recorded at 20°C. Data are shown to the point up to which the dyna voltage was in the permissible range.

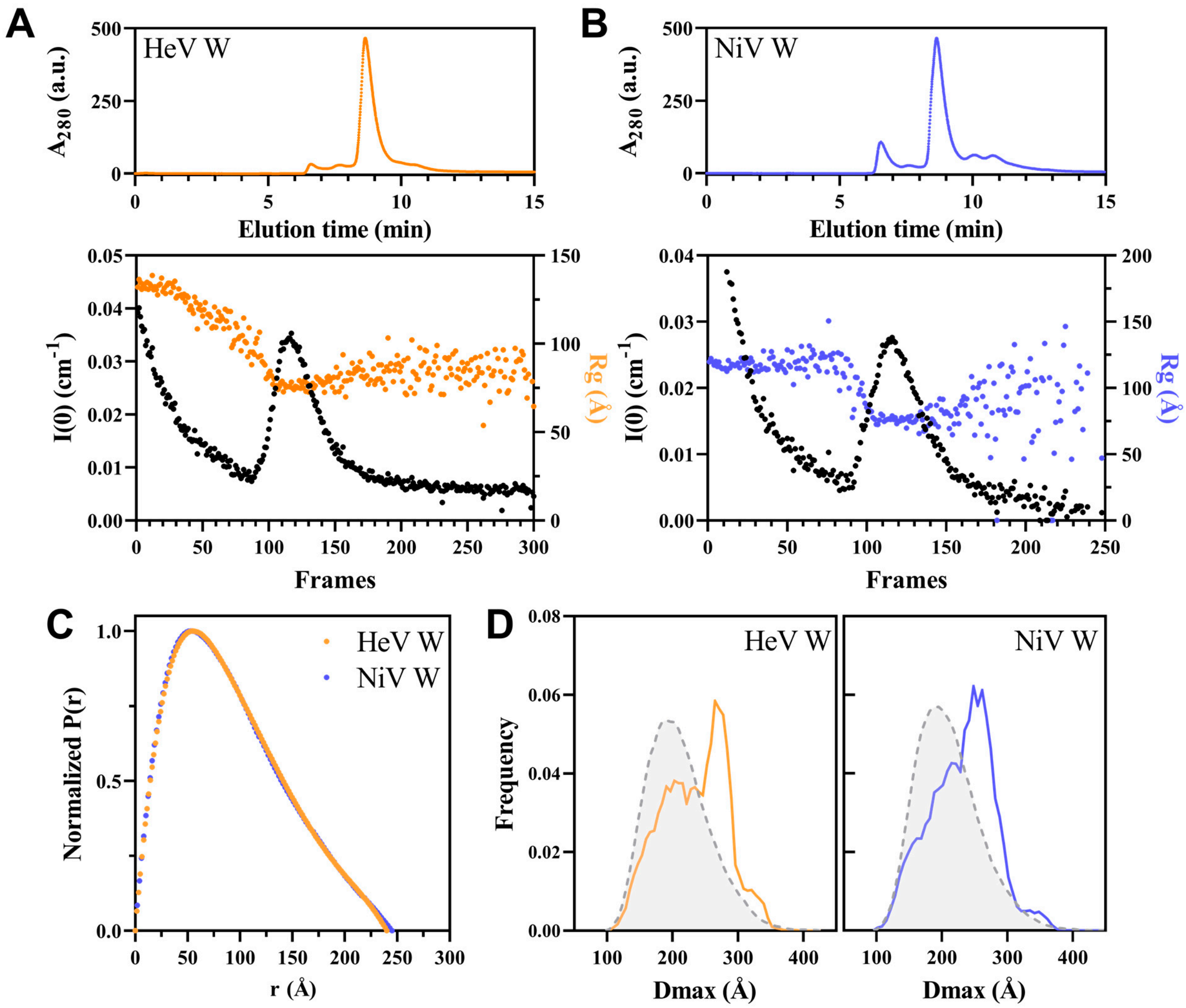

Figure S6. (**A, B**) SEC profile (top) and scattered intensities (bottom) as a function of acquisitions as obtained in SEC-SAXS experiments of the HeV (**A**) and NiV (**B**) W proteins. The estimated R_g *per* frame is shown in the bottom panels. (**C**) Pairwise distance distribution function for the HeV and NiV W proteins. (**D**) D_{max} distribution in the initial pool (dashed grey line) and in the final EOM ensemble of HeV (orange line) and NiV (blue line) W.

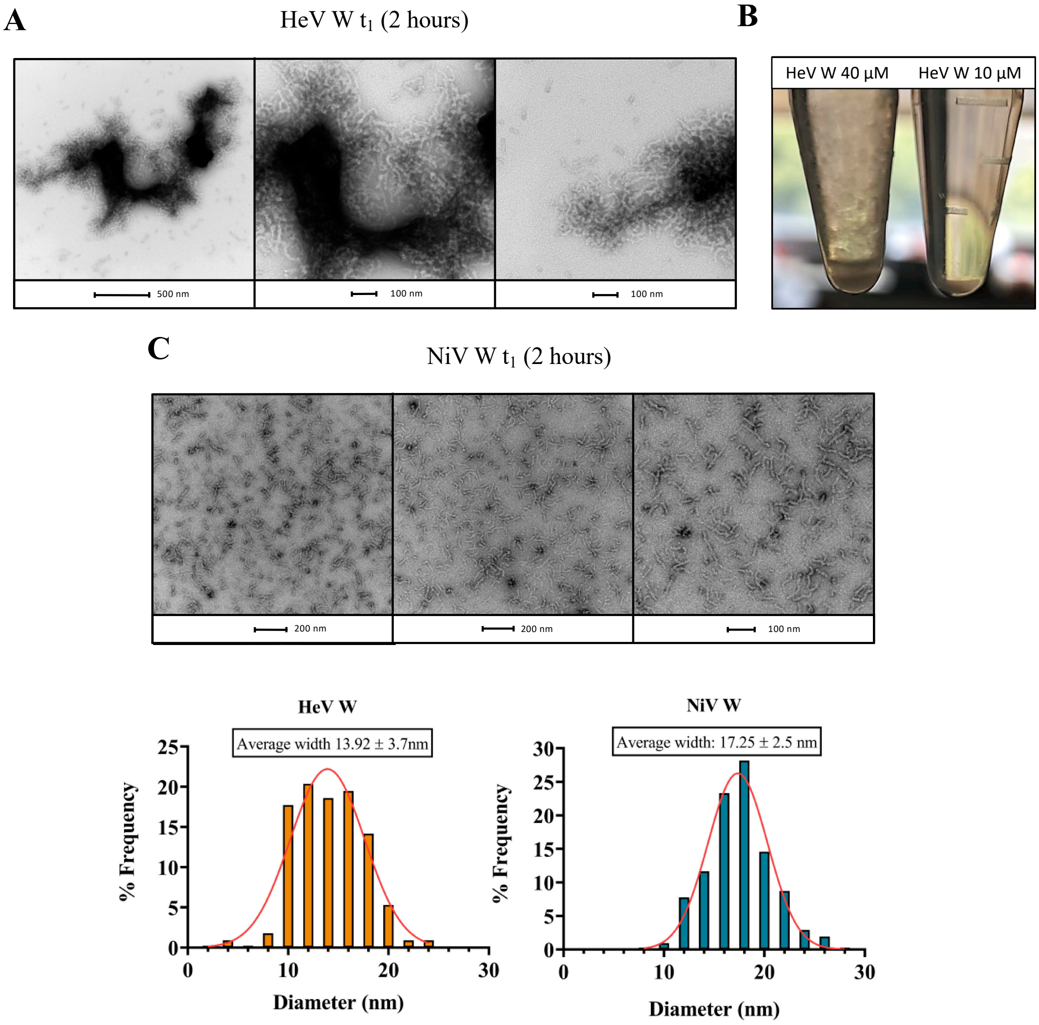

Figure S7. (A) Macroscopically visible aggregates of HeV W formed at two different concentrations upon incubation at 37 °C for 10 hours. **(B, C)** Negative-staining TEM of a HeV (**B**) or NiV (**C**) W sample at 40 µM at t1 (2 hours of incubation) showing the presence of large aggregates. Fibrils formed by NiV W are longer compared to t0 and mono-dispersed on the grid, in contrast to HeV W. **(D)** Distribution of fibril width of HeV and NiV W protein, of which the Gaussian mean is shown above. The two average values are quite close to each other and consistent with the expected size for amyloid-like fibrils [3]. Each bar in the histogram, centered on *n*, corresponds to fibrils whose thickness is comprised between *n*-4 and *n*+5. The analysis was done using the ImageJ software.

Supplementary Table S1. List of partners of the HeV and NiV W proteins. All of the reported partners are human, except for the murine inhibitor of nuclear factor kappa-B kinase subunit alpha protein (UniProt Accession number Q60680), whose sequence is very similar to that of its human counterpart (UniProt Accession number O15111).

W proteins	Protein partners			
	UniProt KB accession number	Gene name	Protein name	Reference (Pubmed ID)
HeVW (P0C1C6)	P04083	ANXA1	Annexin A1	22810585
	Q13185	CBX3	Chromobox protein homolog 3	22810585
	P12277	CKB	Creatine kinase B-type	22810585
	P08311	CTSG	Cathepsin G	22810585
	P59665	DEFA1	Neutrophil defensin 1	22810585
	P59666	DEFA3	Neutrophil defensin 3	22810585
	P12838	DEFA4	Neutrophil defensin 4	22810585
	Q9UGM3	DMBT1	Deleted in malignant brain tumors 1 protein	22810585
	P08246	ELANE	Neutrophil elastase	22810585
	P04406	GAPDH	Glyceraldehyde-3-phosphate dehydrogenase	22810585
	P0C0S8	H2AC11	Histone H2A type 1	22810585
	Q96KK5	H2AC12	Histone H2A type 1-H	22810585
	Q99878	H2AC14	Histone H2A type 1-J	22810585
	Q6FI13	H2AC18	Histone H2A type 2-A	22810585
	Q16777	H2AC20	Histone H2A type 2-C	22810585
	P04908	H2AC4	Histone H2A type 1-B/E	22810585
	Q93077	H2AC6	Histone H2A type 1-C	22810585
	P20671	H2AC7	Histone H2A type 1-D	22810585
	Q9BTM1	H2AJ	Histone H2A.J	22810585
	Q7L7L0	H2AW	Histone H2A type 3	22810585
	O60814	H2BC12	Histone H2B type 1-K	22810585
	Q99880	H2BC13	Histone H2B type 1-L	22810585
	Q99879	H2BC14	Histone H2B type 1-M	22810585
	Q99877	H2BC15	Histone H2B type 1-N	22810585
	Q5QNW6	H2BC18	Histone H2B type 2-F	22810585
	P62807	H2BC4	Histone H2B type 1-C/E/F/G/I	22810585
	P58876	H2BC5	Histone H2B type 1-D	22810585
	Q93079	H2BC9	Histone H2B type 1-H	22810585
	P62805	H4C1	Histone H4	22810585
	P01876	IGHA1	Immunoglobulin heavy constant alpha 1	22810585
	P01857	IGHG1	Immunoglobulin heavy constant gamma 1	22810585
	P01859	IGHG2	Immunoglobulin heavy constant gamma 2	22810585
	P01834	IGKC	Immunoglobulin kappa constant	22810585
	P0CG04	IGLC1	Immunoglobulin lambda constant 1	22810585
	P0DOY2	IGLC2	Immunoglobulin lambda constant 2	22810585
	P0DOY3	IGLC3	Immunoglobulin lambda constant 3	22810585
	P0CF74	IGLC6	Immunoglobulin lambda constant 6	22810585
	O00505	KPNA3	Importin subunit alpha-4	22810585
	O00629	KPNA4	Importin subunit alpha-3	22810585
	Q14974	KPNB1	Importin subunit beta-1	22810585
	P02788	LTF	Lactotransferrin	22810585
	P61626	LYZ	Lysozyme C	22810585
	P05164	MPO	Myeloperoxidase	22810585
	P07737	PFN1	Profilin-1	22810585
	P12273	PIP	Prolactin-inducible protein	22810585
	P24158	PRTN3	Myeloblastin	22810585
	P42224	STAT1	Signal transducer and activator of transcription 1-alpha/beta	22810585
	P52630	STAT2	Signal transducer and activator of transcription 2	22810585
	P31946	YWHAB	14-3-3 protein beta/alpha	22810585
	P62258	YWHAE	14-3-3 protein epsilon	22810585
	P61981	YWHAG	14-3-3 protein gamma	22810585
	P27348	YWHAQ	14-3-3 protein theta	22810585
	P63104	YWHAZ	14-3-3 protein zeta/delta	22810585

W proteins	Protein partners			
	UniProt KB accession number	Gene name	Protein name	Reference (Pubmed ID)
NiVW (P0C1C7)	O75934	BCAS2	Pre-mRNA-splicing factor SPF27	28904190
	Q99459	CDC5L	Cell division cycle 5-like protein	28904190
	Q60680	Chuk	Inhibitor of nuclear factor kappa-B kinase subunit alpha	24269682
	P12277	CKB	Creatine kinase B-type	22810585
	Q02539	H1-1	Histone H1.1	22810585
	P22492	H1-6	Histone H1t	22810585
	P0C0S8	H2AC11	Histone H2A type 1	22810585
	Q96KK5	H2AC12	Histone H2A type 1-H	22810585
	Q99878	H2AC14	Histone H2A type 1-J	22810585
	Q6FI13	H2AC18	Histone H2A type 2-A	22810585
	Q16777	H2AC20	Histone H2A type 2-C	22810585
	P04908	H2AC4	Histone H2A type 1-B/E	22810585
	Q93077	H2AC6	Histone H2A type 1-C	22810585
	P20671	H2AC7	Histone H2A type 1-D	22810585
	Q9BTM1	H2AJ	Histone H2A.J	22810585
	Q7L7L0	H2AW	Histone H2A type 3	22810585
	P62805	H4C1	Histone H4	22810585
	P22626	HNRNPA2B1	Heterogeneous nuclear ribonucleoproteins A2/B1	22810585
	P31942	HNRNPH3	Heterogeneous nuclear ribonucleoprotein H3	22810585
	P11021	HSPA5	Endoplasmic reticulum chaperone BiP	22810585
	O00505	KPNA3	Importin subunit alpha-4	28904190
	O00629	KPNA4	Importin subunit alpha-3	28904190
	Q14974	KPNB1	Importin subunit beta-1	22810585
	P12036	NEFH	Neurofilament heavy polypeptide	22810585
	Q6T4R5	NHS	Nance-Horan syndrome protein	22810585
	O43660	PLRG1	Pleiotropic regulator 1	28904190
	Q9UMS4	PRPF19	Pre-mRNA-processing factor 19	28904190
	P31947	SFN	14-3-3 protein sigma	32321809
	P42224	STAT1	Signal transducer and activator of transcription 1-alpha/beta	28904190
	P52630	STAT2	Signal transducer and activator of transcription 2	22810585
	Q14765	STAT4	Signal transducer and activator of transcription 4	28904190
	P31946	YWHAB	14-3-3 protein beta/alpha	22810585
	P62258	YWHAE	14-3-3 protein epsilon	22810585
	P61981	YWHAG	14-3-3 protein gamma	22810585
	Q04917	YWHAH	14-3-3 protein eta	32321809
	P27348	YWHAQ	14-3-3 protein theta	22810585
	P63104	YWHAZ	14-3-3 protein zeta/delta	22810585

References

1. Campen, A.; Williams, R.M.; Brown, C.J.; Meng, J.; Uversky, V.N.; Dunker, A.K. TOP-IDP-scale: a new amino acid scale measuring propensity for intrinsic disorder. *Protein Pept Lett* **2008**, *15*, 956-963, doi:10.2174/092986608785849164.
2. Lieutaud, P.; Canard, B.; Longhi, S. MeDor: a metaserver for predicting protein disorder. *BMC Genomics* **2008**, *9*, S25.
3. Boyer, D.R.; Mynhier, N.A.; Saway, M.R. Why amyloid fibrils have a limited width. *BioRxiv* **2021**, doi:10.1101/2021.07.02.450971.
